# *Caenorhabditis elegans* N-glycan Core β-galactoside Confers Sensitivity towards Nematotoxic Fungal Galectin CGL2

**DOI:** 10.1371/journal.ppat.1000717

**Published:** 2010-01-08

**Authors:** Alex Butschi, Alexander Titz, Martin A. Wälti, Vincent Olieric, Katharina Paschinger, Katharina Nöbauer, Xiaoqiang Guo, Peter H. Seeberger, Iain B. H. Wilson, Markus Aebi, Michael O. Hengartner, Markus Künzler

**Affiliations:** 1 Institute of Molecular Biology, University of Zürich, Zürich, Switzerland; 2 Institute of Microbiology, Swiss Federal Institute of Technology (ETH) Zürich, Zürich, Switzerland; 3 Swiss Light Source (SLS), Paul-Scherrer-Institute (PSI), Villigen, Switzerland; 4 Department of Chemistry, University of Natural Resources and Applied Life Sciences (BOKU), Vienna, Austria; 5 VetOMICS Core Facility for Proteomics & Metabolomics Studies, University of Veterinary Medicine, Vienna, Austria; 6 Department of Chemistry and Applied Biosciences, ETH Zürich, Zürich, Switzerland; University of Birmingham, United Kingdom

## Abstract

The physiological role of fungal galectins has remained elusive. Here, we show that feeding of a mushroom galectin, *Coprinopsis cinerea* CGL2, to *Caenorhabditis elegans* inhibited development and reproduction and ultimately resulted in killing of this nematode. The lack of toxicity of a carbohydrate-binding defective CGL2 variant and the resistance of a *C. elegans* mutant defective in GDP-fucose biosynthesis suggested that CGL2-mediated nematotoxicity depends on the interaction between the galectin and a fucose-containing glycoconjugate. A screen for CGL2-resistant worm mutants identified this glycoconjugate as a Galβ1,4Fucα1,6 modification of *C. elegans* N-glycan cores. Analysis of N-glycan structures in wild type and CGL2-resistant nematodes confirmed this finding and allowed the identification of a novel putative glycosyltransferase required for the biosynthesis of this glycoepitope. The X-ray crystal structure of a complex between CGL2 and the Galβ1,4Fucα1,6GlcNAc trisaccharide at 1.5 Å resolution revealed the biophysical basis for this interaction. Our results suggest that fungal galectins play a role in the defense of fungi against predators by binding to specific glycoconjugates of these organisms.

## Author Summary

Fungi are a source of a large variety of carbohydrate-binding proteins (lectins). Synthesis of these proteins usually occurs in the cytoplasm and is often restricted to the reproductive organs (fruiting bodies, sclerotia) of the respective fungi. Although these lectins can be very abundant in these organs, their function is unknown. The specificity for non-fungal carbohydrates and recent functional studies in genetically amenable fungi argue against an endogenous function in development. Here we show that oral administration of the fruiting-body-specific galectins of the ink cap mushroom *Coprinopsis cinerea* is toxic for the model nematode *Caenorhabditis elegans* and that the nematotoxicity of these fungal lectins is dependent on binding to a specific β-galactoside occurring on nematode, but not on fungal, N-glycans. Since fungal-feeding nematodes represent the predominant predators of fungi in the soil, these results suggest that these lectins are effectors of a protein-mediated fungal defense system. Lectin-mediated defense strategies against predators, parasites and pathogens are also used by plants and animals. Due to the conservation of this type of innate defense amongst eukaryotes and the reduced complexity of fungi, studies of this in fungi could contribute to a better understanding of analogous systems and the evolution of multi-level defense in animals.

## Introduction

Lectins are defined as non-immunoglobulin, carbohydrate-binding proteins without catalytic activity towards the recognized carbohydrate [1]. They occur in all domains of life and are very diverse with respect to structure, carbohydrate-binding specificity and function [2]. Specific lectins play an important role in the defense against predators, parasites and pathogens due to their ability to recognize specific carbohydrate signatures displayed on the cell surfaces of these organisms. They act either as direct effectors or as opsonins, i.e., by recruiting other effectors of the immune system. Examples of the former type are bacterial toxins directed against bacterivores (*Bacillus thuringiensis* crystal toxins), plant lectins directed against herbivores (*Ricinus communis* (Ricin) toxin), and mammalian lectins directed against bacterial and fungal mucosal pathogens (RegIIIγ [3] and galectin-3 [4], respectively). The effector role of all these lectins is based on their toxicity towards the predator or pathogen. In only a few cases is the molecular basis of this toxicity known in terms of identification of the target glycoconjugate in the predator/pathogen recognized by the lectin. For example, the nematicidal crystal toxin Cry5B from *B. thuringiensis* was shown to bind a specific glycosphingolipid on the intestinal epithelium of the bacterivorous nematode *Caenorhabditis elegans* [5] and antifungal mammalian galectin-3 was shown to bind β1,2-linked mannans on the surface of *Candida albicans* cells [4]. It should be noted here that Ricin and many bacterial toxins contain additional domains with pore-forming or catalytic activity which contribute to the toxicity of these lectins [6].

Fungal fruiting-bodies are a rich source for lectins of various structures and specificities [7],[8],[9],[10]. A specific subgroup of fungal lectins, the so-called fruiting-body lectins, are characterized by their specific expression and abundance in the fruiting body and their cytoplasmic subcellular localization. The latter property is concluded from the absence of a signal peptide for classical secretion and the concomitant lack of disulphide bridges or protein glycosylation. The physiological function of these fruiting body lectins, however, remains unknown. A suggested role in fruiting body formation, based on the developmental regulation of their expression, has become unlikely based on recent studies in the ascomycete (sac fungus) *Sordaria macrospora* and the homobasidiomycete (mushroom) *Coprinopsis cinerea* [11],[12]. Given the frequent role of lectins in defense of other organisms and the reported toxicity of fruiting-body lectins towards insects and mammalian cells [13],[14],[15],[16],[17],[18],[19], we and others hypothesized that this group of lectins may be effectors of a fungal defense system against fungivores. Examples for predators that have specialized on fungi as food source are larvae of some flies and fungal-feeding nematodes [20],[21],[22]. In comparison to insects, fungal-feeding nematodes represent, due to their high number in soil and any kind of decaying organic matter, a major threat to fungi in their ecological environment [23],[24].

The fungus *C. cinerea* expresses several fruiting body lectins including two isogalectins, CGL1 and CGL2, and a galectin-related lectin, CGL3 [25],[26],[27]. In agreement with above hypothesis, we demonstrate that both *C. cinerea* isogalectins displayed toxicity towards the model soil nematode *C. elegans*. Based on a number of approaches, including the use of *E. coli* expressing these fungal lectins to mimic the *in vivo* situation, we conclude that this nematotoxicity is dependent on the specific interaction between these proteins and a β-galactoside on the core of N-glycans in the *C. elegans* intestine.

## Results

### CGL2 inhibits *C. elegans* development and reproduction dependent on its carbohydrate-binding ability

**Figure 1:**
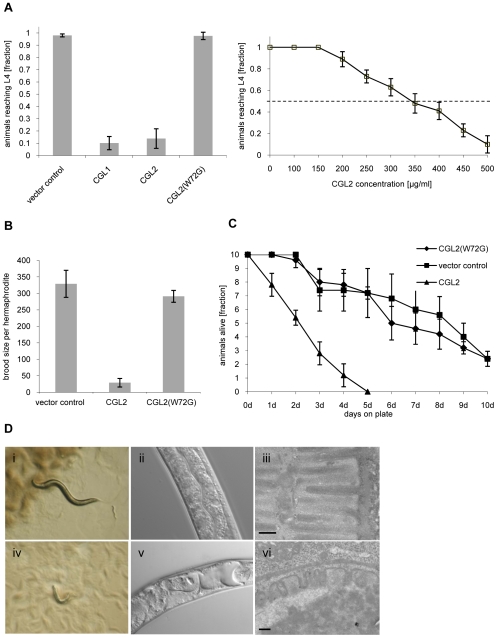
Dose and carbohydrate-binding dependent toxicity of *C. cinerea* galectin CGL2 towards *C. elegans*. In all of the experiments, *E. coli* BL21(DE3) cells expressing either the authentic CGL1 or CGL2 proteins or the carbohydrate-binding defective variant CGL2(W72G), or control transformants were fed to *C. elegans* wild type N2. (A) CGL2 inhibits *C. elegans* development. *C. elegans* L1 larvae were seeded onto lawns of above bacteria (left panel) or fed with increasing concentrations of purified CGL2 together with equal amounts of empty vector-containing BL21(DE3) in liquid culture (right panel) and scored for the fraction developing to the L4 stage within 72 h and 96 h, respectively. Columns represent the average of 6 and 12 replicates, respectively. Error bars indicate standard deviations. The fraction of animals reaching L4 was significantly lower on CGL1- or CGL2-expressing bacteria (p<0.01) than on bacteria expressing CGL2(W72G) or containing empty vector. No significant difference was observed between latter two conditions (p>0.5). In the liquid assay, worm development decreases significantly at CGL2 concentrations higher than 150 µg/ml. (B) CGL2 inhibits *C. elegans* reproduction. *C. elegans* hermaphrodites were placed as L4 animals on plates seeded with above bacteria and scored for total progeny counts per hermaphrodite. The broods of eight to ten hermaphrodites were averaged per data point. The standard deviations are indicated. The differences between the L4 fractions on CGL2 and vector control and CGL2(W72G) were statistically significant (p>0.01). None of the progeny on wild type CGL2 developed to adulthood within 96 h post hatch. (C) CGL2 ultimately kills *C. elegans*. 10 L4 staged wild type *C. elegans* (N2) were seeded onto lawns of CGL2-, CGL2(W72G)-expressing and empty vector control-containing *E. coli* BL21(DE3) cells. Each day, the plates were checked for surviving animals which were then transferred to novel bacterial lawns of the same type. The data points represent the average of five replicates. Error bars indicate standard deviations. The survival rate of the worms was significantly impaired on CGL2-expressing bacteria compared to CGL2(W72G) and vector control (p<0.01), whereas there was no significant difference between latter two conditions (p>0.5). (D) CGL2 damages *C. elegans* intestine. *C. elegans* L4 larvae were fed with CGL2-expressing (panels i-vi) and control *E. coli* BL21(DE3) cells (panels i-iii) and examined after 24 h under the stereomicroscope (panels i,iv), by differential interference contrast (DIC) microscopy (panels ii, v) and by transmission electron microscopy (TEM) (panels iii,vi). The size bars in panels iii and vi are 200 nm.

To test the *C. cinerea* galectins for nematotoxic activity in a genetically tractable system, the bacterivorous model nematode *C. elegans* was fed with *E. coli* cells expressing either the authentic CGL1 and CGL2 proteins or the carbohydrate-binding defective variant CGL2(W72G) in the cytoplasm in comparison to ‘empty vector’-containing *E. coli* transformants. The essential role of W72 in the coordination of β-galactosides by CGL2 was demonstrated previously [28]. For CGL2 and CGL2(W72G), the effect of this diet on *C. elegans* development, reproduction and survival was examined by determining the fraction of L1 larvae developing to the L4 stage within 72 h, the brood size per hermaphrodite and the survival of L4 larvae, respectively. The results of all three assays showed a strong effect of the fungal galectin, i.e., the animals that fed on CGL2-expressing bacteria were severely inhibited in larval development and showed a reduced brood size and survival (Fig. 1, panels A to C). The nematotoxicity was dependent on the ability of CGL2 to bind carbohydrates since the CGL2(W72G) variant, which does not possess β-galactoside binding activity [28], did not show any effect in either assay. For CGL1, only larval development was scored, yielding similar results as in the case of CGL2 (Fig. 1A, left panel).

The effect of using bacteria expressing CGL2 was verified using the purified recombinant lectin. Incubation of L1 larvae with different concentrations of purified CGL2 with empty vector-containing bacteria demonstrated that the lectin-mediated arrest in larval development was dose-dependent with a median lethal dose (LD_50_) of approximately 350 µg/ml (Fig. 1, panel A right). Comparative light- and electron-microscopy of *C. elegans* L4 larvae fed with CGL2-expressing or empty vector-containing *E. coli* cells revealed that the lumen of the anterior intestine was expanded in the former animals and that the microvillar ultrastructure of the epithelium was damaged (Fig. 1, panel D). These results demonstrated that the *C. cinerea* galectin CGL2 had a pronounced nematotoxic activity that was dependent on the ability of the protein to bind carbohydrates.

### The CGL2-ligand in *C. elegans* is different from the Cry5B-ligand but its biosynthesis also relies on fucose biosynthesis

**Figure 2:**
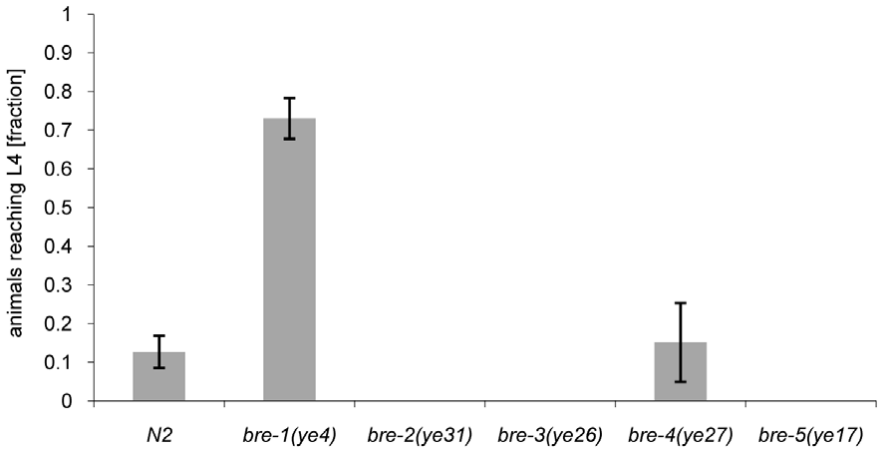
Resistance of *bre-1* mutant towards CGL2-mediated toxicity. L1 larvae of *C. elegans bre-1* to *bre-5* mutant strains as well as wild-type N2 were seeded onto a lawn of CGL2-expressing *E. coli* BL21(DE3) cells and scored for the fraction developing to the L4 stage within 72 h. Columns represent the average of 6 replicates. Error bars indicate standard deviations. The fraction of animals reaching L4 was significantly higher for the *bre-1* mutant (p<0.01) than for the wild type (N2) or the *bre-4* mutant. No significant difference was observed between latter two strains (p>0.5). For the rest of the mutants, not a single larva developed, so results were not compared statistically.

The phenotype of CGL2-mediated nematotoxicity was reminiscent of the intoxication of *C. elegans* induced by feeding with *E. coli* cells expressing the nematicidal pore-forming crystal toxin Cry5B from *Bacillus thuringiensis* [29]. Indicative of the role of carbohydrate binding in their function, crystal toxins contain lectin domains that are necessary for toxicity [30] and indeed a genetic screen for Cry5B-resistant *C. elegans* mutants yielded mutations in five different genes, *bre-1(ye4)*, *bre-2(ye31)*, *bre-3(ye26)*, *bre-4(ye27)* and *bre-5(ye17)* [29]. Four of these *C. elegans* genes code for glycosyltransferases involved in the biosynthesis of a specific, β-galactoside-containing glycosphingolipid (*bre-2* to *bre-5*) and one gene encodes an enzyme involved in the conversion of GDP-mannose into GDP-fucose (*bre-1*) [31]. Given the specificity of *C. cinerea* galectins for β-galactoside-containing oligosaccharides [27],[28], we tested each *bre* mutant for resistance to CGL2. Unexpectedly, we found that the *bre-1* mutation conferred almost complete resistance whereas the other *bre* mutants, with the exception of the *bre-4* mutant, that was as sensitive as wild type *C. elegans*, appeared even more sensitive to CGL2 than wild type (Fig. 2). The *bre-1(ye4)* mutant also showed resistance towards CGL1-mediated nematotoxicity suggesting that CGL1 acts via the same mechanism (data not shown). These results demonstrated that the ligand of *C. cinerea* galectins in *C. elegans* was different from the Cry5B-ligand but that its biosynthesis relied on fucose biosynthesis as in the case of the bacterial toxin.

### Mutations in the *C. elegans* p38 MAPK pathway cause hypersensitivity to CGL2

**Figure 3:**
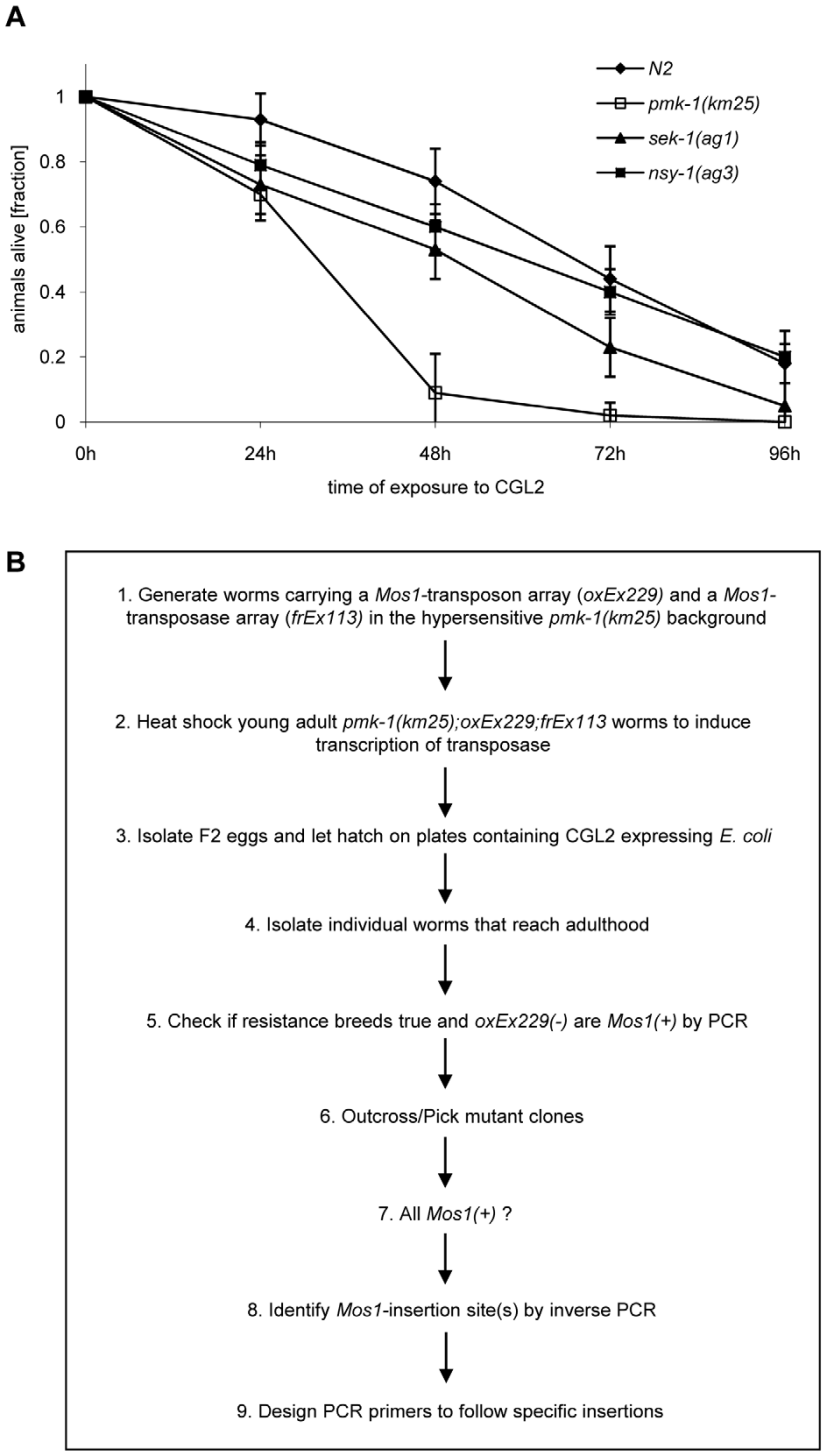
Workflow of the forward genetic screen for CGL2-resistant *C. elegans* mutants. (A) CGL2-sensitivity test of *pmk-1*, *sek-1* and *nsy-1* mutant worms defective in the p38 MAPK pathway. 10 L4 staged worms of the indicated genotypes were seeded onto a lawn of CGL2-expressing *E. coli* BL21(DE3) cells. The plates were checked for surviving animals at the indicated time points. The data points represent the average of ten replicates. Error bars indicate standard deviations. The *pmk-1* and *sek-1* mutants were significantly hypersensitive compared to the N2 wild type strain (p<0.01), whereas the slightly higher sensitivity of the *nsy-1* mutant is less significant (p<0.2). (B) *Mos1* insertional mutagenesis workflow. Worms that carry the *Mos1* transposon array *oxEx229* and the *Mos1* transposase array *frEx113* in the CGL2-hypersensitive *pmk-1(km25)* background were generated.

**Figure 4:**
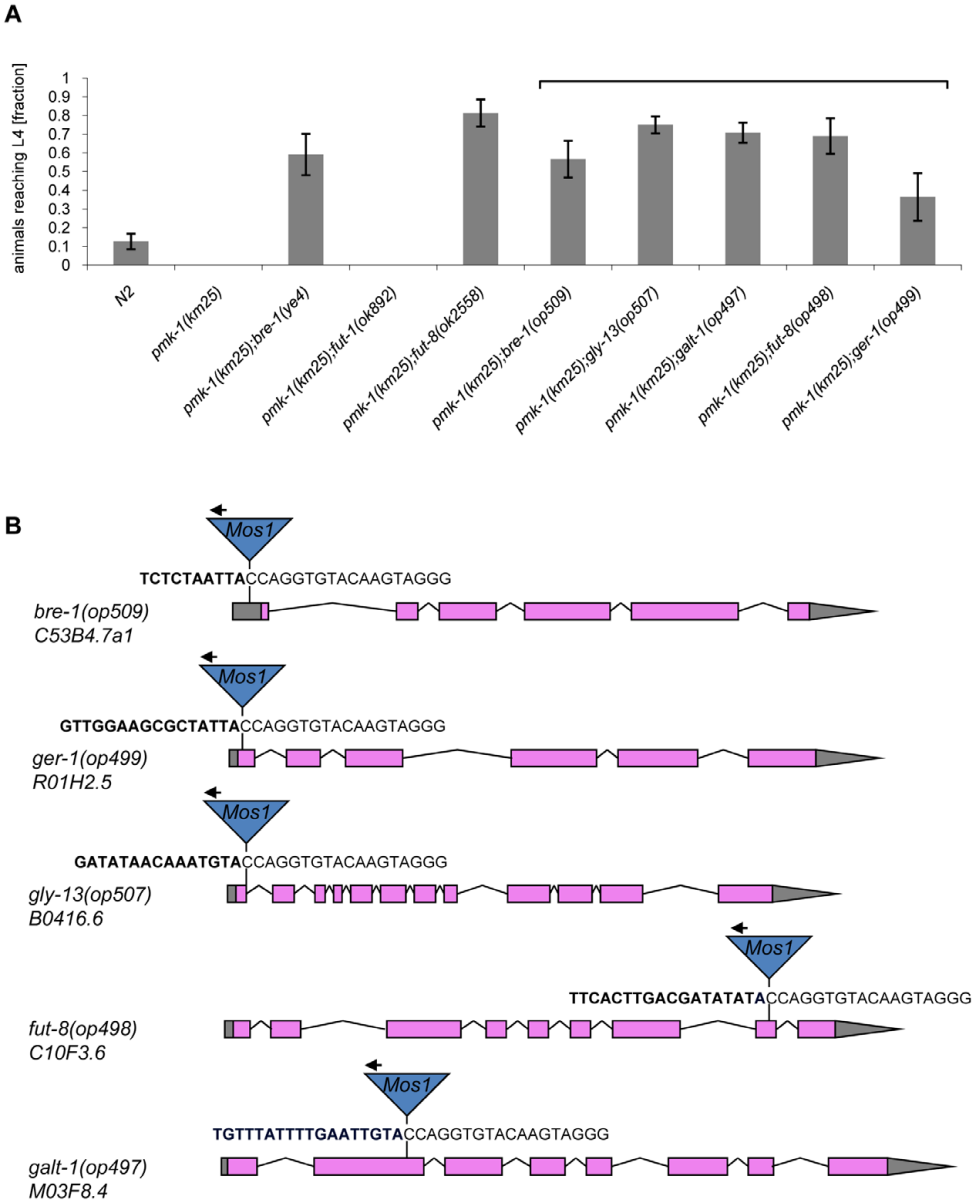
Results of the forward genetic screen for CGL2-resistant *C. elegans* mutants. (A) Resistance of isolated and constructed *C. elegans* mutants towards CGL2-mediated toxicity. *C. elegans* mutants of the indicated genotypes were analysed for development from L1 to L4 as outlined above. The gene appendix *(op)* and the bracket above the histogram indicate mutants isolated in the *Mos1*-screen. The other mutants were constructed by crossing. The increase in the fractions of animals reaching L4 of the various mutants compared to N2 worms was statistically significant (p<0.05). In case of the *pmk-1* and the *pmk-1;fut-1* mutant, not a single larva developed, so results were not compared statistically. (B) Insertion sites of *Mos1* elements in CGL2-resistant mutants. Arrows above *Mos1* elements indicate the orientation of the *Mos1* primer oJL115 used for sequencing iPCR products of mutant lysates. Bold letters indicate *C. elegans* genomic sequences and are followed by *Mos1* sequence. Gene models are taken from WormBase Release WS207. *bre-1(op509)* mutants have a *Mos1* insertion in the 5′-UTR of *C53B4.7a1*, located 184 bp upstream of the translational start codon. *ger-1(op499)* mutants have a *Mos1* insertion in the first exon of *R01H2.5,* located 50 bp downstream of the translational start codon. *gly-13(op507)* mutants have a *Mos1* insertion in a conserved splicing donor site flanking the first exon of *B0416.6*. *fut-8(op498)* mutants have a *Mos1* insertion in the eighth exon of *C10F3.6. galt-1(op497)* mutants have a *Mos1* insertion in the second exon of *M03F8.4.*

Based on the resistance of the *C. elegans bre-1(ye4)* mutant towards feeding of CGL2-expressing bacteria we initiated a forward genetic screen for mutations conferring CGL2-resistance in order to identify the target glycoconjugate of CGL2 in *C. elegans*. For an efficient selection of CGL2-resistant mutants, it was essential to lower the background of 10% surviving worms in case of *C. elegans* wild type strain (N2) (Fig. 4, panel A). Since mutants in the p38 mitogen-activated protein kinase (MAPK) pathway of *C. elegans* were previously shown to be hypersensitive towards pathogenic bacteria and Cry5B-expressing *E. coli* cells [32],[33], we tested whether these strains would also be more susceptible towards feeding with the CGL2-expressing *E. coli* cells. For this purpose, L4 larvae derived from N2 wild type and isogenic *pmk-1(km25)* (p38 MAPK), *sek-1(ag1)* (MAPK kinase) and *nsy-1(ag3)* (MAPK kinase kinase) mutant worms were tested for survival on a lawn of CGL2-expressing bacteria over time. We found that all of the mutations increased the sensitivity of *C. elegans* towards the fungal galectin but that the effect of the *pmk-1* mutation was most pronounced (Fig. 3, panel A). As a proof of principle for the subsequent screen, we constructed a worm carrying the *bre-1(ye4)* mutation in a *pmk-1(km25)* mutant genetic background and tested this strain for its sensitivity to CGL2 in comparison to the *pmk-1(km25)* single mutant strain. The double mutant strain exhibited significant resistance towards CGL2 confirming the previous results in the wild type background and suggesting that a forward genetic screen for mutations conferring CGL2-resistance in a *pmk-1(km25)* mutant genetic background was feasible (Fig. 4, panel A). The screen, as outlined in Fig. 3 (panel B), was based on an insertional mutagenesis using the *Mos1* transposon to facilitate the identification of the obtained mutations.

### Mutations affecting α1,6-core fucosylation of *C. elegans* N-glycans confer resistance to CGL2 and prevent binding of CGL2 to the intestine

The screen yielded *Mos1*-insertions in five different genes: *bre-1*, *ger-1*, *gly-13*, *fut-8* and *M03F8.4*. The degree of resistance towards CGL2-intoxication was determined for each of the outcrossed mutants and is shown in Fig. 4 (panel A). The identified *Mos1*-insertions are located in 5′-untranslated regions (*bre-1(op509)*), at an exon-intron junction (*gly-13(op507)*) and in exons (*ger-1(op499)*, *fut-8(op498)* and *M03F8.4(op497)*), respectively (Fig. 3, panel D). *bre-1* and *ger-1* code for the GDP-mannose-4,6-dehydratase and GDP-4-keto-6-deoxymannose-3,5-epimerase-4-reductase, respectively, catalyzing the two enzymatic steps in the conversion from GDP-mannose to GDP-fucose [34]. The link between the biosynthesis of GDP-fucose and the biosynthesis of a specific glycoconjugate was offered by the data indicating a role for the *fut-8* and *gly-13* genes in toxicity, suggesting that N-glycans are the targets of CGL2. The *fut-8* gene encodes the only fucosyltransferase capable of transferring fucose in α1,6-configuration from GDP-fucose to the asparagine-linked GlcNAc in *C. elegans* N-glycan cores [35], whereas the *gly-13* gene codes for the major GlcNAc-transferase I (GnTI) in *C. elegans* [36], which transfers GlcNAc in β1,2-configuration to the α1,3-linked mannose of pentamannosidic N-glycan. The modification of N-glycans by GnTI is the prerequisite for many of the subsequent modifications of N-glycans including the action of core α1,6-fucosyltransferase [37]; on the other hand, GnTI is not required in *C. elegans* for the core modification by another fucosyltransferase, encoded by the *fut-1* gene, which forms the so-called HRP epitope consisting of fucose α1,3-linked to the core asparagine-linked GlcNAc [38].

In order to confirm and corroborate these data, two available mutations affecting both types of core fucosylation, *fut-8(ok2558)* and *fut-1(ok892)*, were each crossed into the *pmk-1* mutant background and the resulting double mutants were tested for CGL2-sensitivity. In agreement with the results of the forward genetic screen, the *fut-8(ok2558)* mutation conferred clear resistance towards CGL2, recapitulating the phenotype shown by the *fut-8(op498)* allele, whereas the *fut-1 pmk-1* double mutant was as susceptible as the *pmk-1* single mutant (Fig. 4, panel A). Analogous tests for CGL1-sensitivity yielded similar results providing additional evidence that CGL1 and CGL2 confer nematotoxicity via the same mechanism (data not shown).

**Figure 5:**
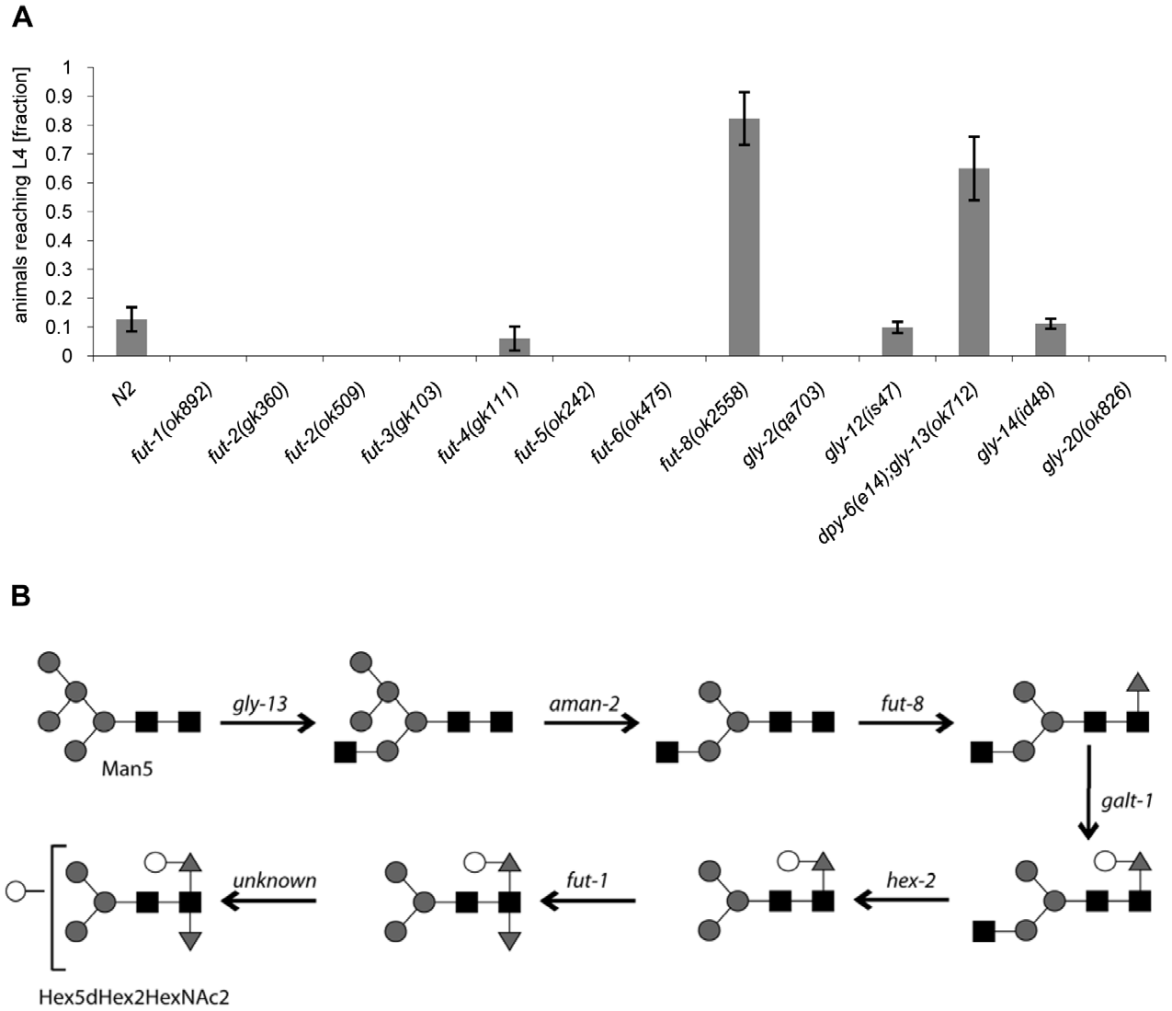
CGL2-sensitivity test and biosynthetic context of available *C. elegans* fucosyltransferase and GlcNAc-transferase mutants. (A) CGL2-sensitivity test. *C. elegans* mutants of the indicated genotypes were analysed for development from L1 to L4 as outlined above. The differences between the L4 fractions of the *fut-4(gk111)* and *dpy-6(e14);gly-13(ok712)* mutants to N2 are statistically significant (p<0.01) in contrast to the differences between the L4 fractions of the *fut8(ok2558)*, *gly-12(is47)* and *gly-14(id48)* mutants to N2 (p<0.9). The differences of the residual mutants to N2 could not be evaluated due to the lack of variance. (B) Biosynthetic context. The putative pathway for biosynthesis of the CGL2 epitope, based on previous *in vivo* and *in vitro* data, indicates the key roles of the enzymes encoded by the *gly-13*, *fut-8* and *galt-1(M03F8.4)* genes; on the other hand, FUT-1 acts after the processing by hexosaminidases such as HEX-2. Other fucosyltransferases such as FUT-2 through to FUT-6 are not involved in modification of the reducing-terminal GlcNAc residue of N-glycans, whereas the *gly-2* and *gly-20* encoded enzymes are not prerequisites for α1,6-fucosylation by FUT-8. Further modifications by other enzymes, encoded by unknown genes, are possible and result in structures such as the depicted Hex_5_dHex_2_HexNAc_2_ glycan.

In addition, available mutants of all other characterized *C. elegans* fucosyltransferase (*fut*)- and N-acetylglucosaminyltransferase (*gly*)-encoding genes were tested for CGL2-sensitivity in comparison to wild type N2 worms. With exception of *fut-8(ok2558)* and *gly-13(ok712)* and consistent with the putative biosynthetic roles of the relevant genes, none of the tested mutations conferred resistance in this background suggesting that the effects of the mutations identified in the screen were highly specific (Fig. 5). In the case of *gly-13*, the tested strain background carried a mutation in an additional gene, *dpy-6*, that is required for normal body morphology but is not involved in glycosylation [39]. Thus, all of the identified genes conferring CGL2-resistance, with the exception of the previously uncharacterized gene *M03F8.4*, suggested a specific role of α1,6-linked fucosylation at the asparagine-linked GlcNAc of *C. elegans* N-glycans in CGL2-mediated nematotoxicity.

**Figure 6:**
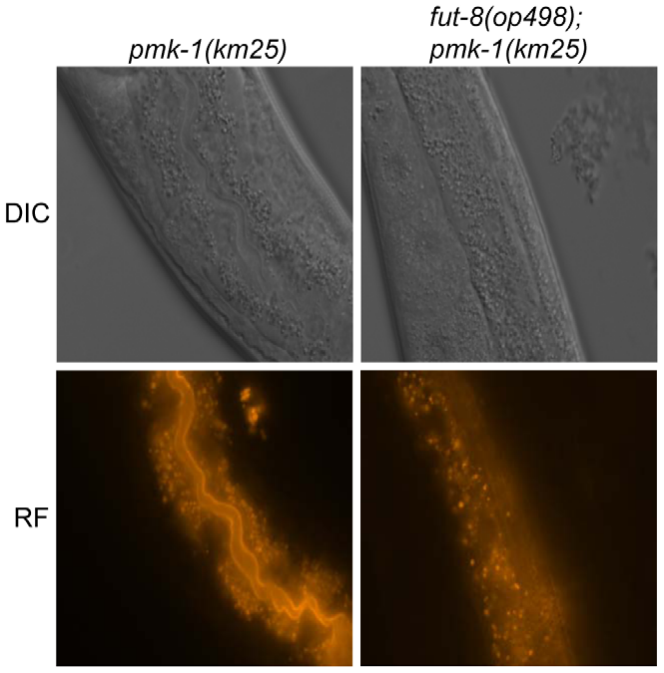
*In situ* localization of the glycoepitope recognized by CGL2. *C. elegans* CGL2-sensitive *pmk-1(km25)* and CGL2-resistant *pmk-1(km25);fut-8(op498)* worms were fed with TAMRA-labeled CGL2 and examined by differential interference contrast (DIC) and red fluorescence (RF) microscopy. 32 and 25 animals, respectively, were scored for staining of the intestinal epithelium (see Supplementary Table S1). The numbers of worms with stained intestinal epithelium was significantly different between the two genetic backgrounds (p<0.01).

To confirm these data and localize the target glycoconjugate of CGL2 *in situ*, L1 larvae of *pmk-1* and *pmk-1;fut-8(op498)* animals were incubated for 24 h in a solution containing tetramethylrhodamine(TAMRA)-labeled CGL2, transferred to plates seeded with standard OP50 bacteria and examined by fluorescence microscopy thereafter. The pictures revealed a distinct red fluorescent signal on the intestinal epithelium of *pmk-1* but not of *pmk-1;fut-8(op498)* animals suggesting that core α1,6-fucosylated N-glycans are the *in vivo* ligands of CGL2 and that this glycoepitope recognized by CGL2 localizes mainly to the intestinal epithelium of *C. elegans* (Supplementary Table S1; Fig. 6).

### Identified *M03F8.4* mutants are defective in galactose capping of α1,6-core fucose in *C. elegans* N-glycans

**Figure 7:**
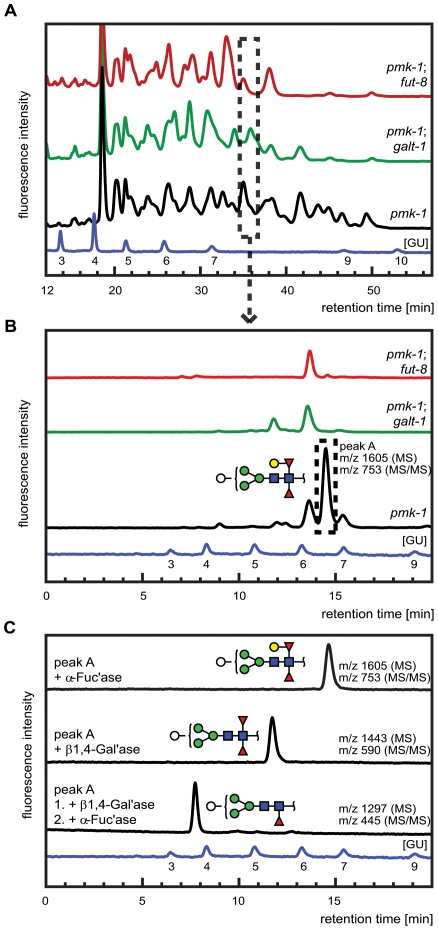
Comparative analysis of the N-glycome in CGL2-resistant *C. elegans* double mutants *pmk-1;fut-8(op498)* (red trace) and *pmk-1(km25);M03F8.4(op497)* (green trace) and the isogenic CGL2-hypersensitive single mutant strain *pmk-1(km25)* (black trace). (A) and (B) HPLC of released and fluorescently labeled N-glycans. Upon enzymatic release (here the PNGase F resistant, but PNGase A sensitive glycome fraction is shown) and fluorescent labeling, N-glycans were separated by normal phase HPLC and analysed by mass spectrometry (A). Fractions at similar retention times (e.g., dashed rectangle) were further separated by reversed phase HPLC and the resulting pure glycans were analysed by mass spectrometry (MS) and for selected fractions by MS/MS (B). (C) Structural characterization of a selected isolated peak. Peak A found in the *pmk-1* strain but not in the two double mutant strains was treated with β1,4-galactosidase (β1,4-Gal'ase) and α-fucosidase (α-Fuc'ase). The reaction products were analysed by reversed phase HPLC and MS and MS/MS (see Supplementary Figure S2). The blue HPLC trace represents the glycan standard in glucose units (GU). Monosaccharides are represented as symbols: Man (green circle), Gal (yellow circle), GlcNAc (blue square), Fuc (red triangle), Hex (white circle).

Based on these results, the specificity of CGL2 for β-galactosides and the reported capping of core α1,6-fucose on *C. elegans* N-glycans with galactose residues in β1,4-configuration [40], we hypothesized that binding of CGL2 to this Galβ1,4Fucα1,6 epitope was required for its nematotoxicity. We surmised that the product of the *M03F8.4* gene, which encodes a putative type II membrane protein with a predicted glycosyltransferase GT-A domain [41] may be, directly or indirectly, involved in the biosynthesis of this N-glycan core modification. In order to validate this hypothesis, the N-glycomes of the *pmk-1* single mutant and the identified CGL2-resistant *pmk-1(km25);fut-8(op498)* and *pmk-1(km25);M03F8.4(op497)* double mutants were analysed. N-glycans were released sequentially by PNGase F and PNGase A to separate core α1,3-fucosylated structures from those devoid of this epitope, labeled with 2-aminopyridine (PA) and analysed by comparative two-dimensional HPLC combined with MALDI-TOF mass spectrometric and enzymatic analysis of the individual glycans. *N*-glycans of the analysed strains showed significant differences in the normal phase 1^st^-dimension chromatograms (Fig. 7, panel A). Among the PNGase F resistant *N*-glycans, i.e. those bearing an α1,3 fucose at the reducing end Glc*N*Ac, peaks eluting at high retention times (t_R_ = 40−50 min) were strongly reduced in the identified M03F8.4 mutant and were completely absent in the identified *fut-8* mutant; such a shift in NP-HPLC chromatograms is consistent with a loss of higher molecular weight glycans.

The individually obtained fractions were subsequently resolved in a second dimension by reversed phase HPLC into pure glycans suitable for structural characterization. The comparison of the 2^nd^ dimension chromatograms of 1^st^ dimension fractions of equal retention times revealed pronounced differences between the *N*-glycomes of the *pmk-1* strain and the two identified CGL2-resistant mutant strains also for the region with less obvious differences in the 1^st^ dimension chromatograms (*i.e.*, t_R_ = 20−40 min). For example, fractions at t_R_ = 34.0−35.5 min in the 1^st^ dimension yielded comparable 2^nd^ dimension patterns (Fig. 7, panel B) with a highly abundant sub-fraction (t_R_ = 14.5 min) being only present in the *pmk-1* pseudo wild type strain. MS analysis identified this peak as a pyridylaminated glycan with the net composition Hex_5_dHex_2_Hex*N*Ac_2_-2-amino-pyridine(PA) (m/z 1605). By MS/MS a daughter ion at m/z 753 was observed, compatible with this glycan containing a chitobiose core carrying α1,3- and α1,6-difucosylation at the reducing end Glc*N*Ac and an additional hexose linked to this reducing end pyridylaminated trisaccharide.

The purified Hex_5_dHex_2_Hex*N*Ac_2_ PA-glycan was subsequently treated with exoglycosidases and the reaction products were further analysed by HPLC and MALDI-TOF MS and MS/MS (Fig. 7, panel C). First, this saccharide was exposed to α-fucosidase which prefers terminal α1,6 linked over α1,3 linked fucoses but no shift in its HPLC elution characteristics was observed (t_R_ = 14.5 min) and the mass was unaltered as determined by mass spectrometry. When this glycan was treated with a fungal (*Aspergillus*) β1,4-specific galactosidase instead, a shift in retention time was observed (t_R_ = 12.5 min) and a loss of one hexose was revealed by MS (*m/z* 1443) and assigned to the hexose linked to the difucosylated reducing-terminal GlcNAc due to the absence of the *m/z* 753 daughter ion in the reaction product. The octasaccharide obtained (t_R_ = 12.5 min) then proved sensitive towards α-fucosidase and loss of the ‘decapped’ terminal α1,6-linked fucose was observed (t_R_ = 7.5 min, *m/z* 1297). These results demonstrated the presence of a β1,4-linked galactose epitope on the α1,6-linked fucose, a modification described previously by Reinhold and co-workers [40]. Analysis of the complete set of 2D-HPLC-MS-MS/MS data (Supplementary Figure S2) confirmed the absence of the galactosylated fucose epitope in the identified *M03F8.4* mutant, suggesting that this gene encodes a glycosyltransferase that is required for the biosynthesis of the β1,4-galactoside linked to the core α1,6- fucose residue. The biochemical characterization of this enzyme, termed GALT-1, is published elsewhere [42]. Based on the available evidence, a pathway for the biosynthesis of Hex_5_dHex_2_HexNAc_2_ can be proposed (Fig. 5, panel B).

### Structural basis for the recognition of Galβ1,4Fuc by CGL2

**Figure 8:**
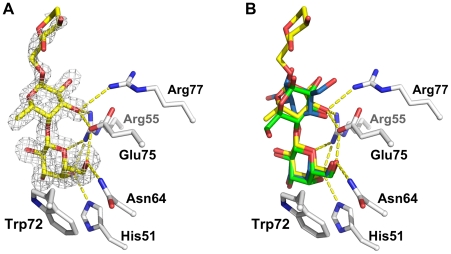
Detailed view of the interaction between CGL2 and Galβ1,4Fucα1,6GlcNAc (A) and comparison with two other CGL2/carbohydrate complexes (B). (A) Fourier difference map (with Fo - Fc coefficients) around the visible part of ligand contoured at 3 σ. Residues belonging to the binding pocket are displayed as sticks and H-bonds as dashed yellow lines. (B) Superimposition of the lactose (Galβ1,4Glc) (green, PDB ID 1ULC) and of the Thomsen-Friedenreich antigen (Galβ1,3GalNAc) (blue, PDB ID 1ULG) onto the CGL2/Galβ1,4Fucα1,6GlcNAc structure (yellow, PDB ID 2WKK). The binding pocket is almost identical in all three structures.

In order to provide biochemical and structural evidence for the interaction between CGL2 and this nematode-specific β-galactoside, the trisaccharide Galβ1,4Fucα1,6GlcNAc was chemically synthesized with a linker at the reducing end and used as ligand in *in vitro* binding experiments with affinity-purified CGL2. Isothermal titration microcalorimetry measurements confirmed binding of this trisaccharide to CGL2 and suggested a dissociation constant of approximately 100 µM (Supplementary Figure S1). The molecular basis of this interaction was investigated by determining the X-ray structure of cocrystals between CGL2 and the trisaccharide at 1.5 Å resolution (Fig. 8, panel A; Supplementary Table S2). Comparisons with the previously determined structures of complexes between CGL2 and the Thomsen-Friedenreich antigen (Galβ1,3GalNAc) and lactose (Galβ1,4Glc) [28] revealed that the structure of CGL2 as well as the direct hydrogen bonds between CGL2 and the β-galactoside of the different carbohydrate ligands were fully superimposable (Fig. 8, panel B). A major difference between these structures lies in the orientation of the preceding monosaccharide subunits to the protein, e.g. the plane of Glc in lactose is perpendicular to the plane of Fuc in the trisaccharide. Due to the low number of direct contacts, there seems to exist a high degree of flexibility on the side of CGL2 with regard to the identity of this monosaccharide subunit. The only constraint is the orientation of the 3′ (in glucose and fucose) or 4′ (in GalNAc) OH of the linked monosaccharide to allow interaction with the conserved carbohydrate-coordinating residues, Arg55 and Glu75. In addition, these hydroxyl groups make additional contact to Arg77 in case of Glc (in lactose) and Fuc (in Galβ1,4Fucα1,6GlcNAc) but not in case of GalNAc (in Thomsen-Friedenreich antigen).

## Discussion

Galectins are β-galactoside-binding lectins known primarily from animals and fungi. In animals, galectins appear to have important roles in development and immunity [43],[44],[45],[46]. The function of the fungal representatives of this lectin family, however, has remained unclear. A role of fungal galectins in fruiting body development, as suggested by their specific expression in these reproductive structures [26] and the identification of a putative endogenous ligand [47] could not be confirmed: Neither constitutive expression nor silencing of the two *C. cinerea* isogalectins, CGL1 and CGL2, affected fruiting body formation [11]. These results together with the toxicity of another mushroom galectin, *Agrocybe aegerita* AAL, towards HeLa cells and mice [14],[48] prompted us to follow the hypothesis of a role of fungal galectins in the defense of higher fungi against predators such as fungal-feeding nematodes. In agreement with this hypothesis, we demonstrate that the *C. cinerea* galectin CGL2 exhibited a pronounced toxicity towards the model nematode *C. elegans*. The nematotoxic activity of CGL2 with a LD_50_ of 350 µg/ml was comparable to the entomotoxic activity of XCL, a fruiting body lectin from another homobasidiomycete and of plant lectins [13].

A role of fungal galectins in defense against predatory nematodes is in line with their predicted cytoplasmic localization, since fungal-feeding as well as plant-feeding nematodes feed by adsorbing the cellular content using a mouth organ called stylet that acts like a syringe [24]. In this light, the cytoplasm, which is essentially free of complex glycoconjugates, is an ideal storage compartment for lectins that are destined to find their ligands in the intestine of predators [Comment: in this study, the anatomical limitation of *C. elegans*, as regards a stylet, is circumvented by expression of the recombinant lectin in the cytoplasm of bacteria]. Indeed, a growing number of cytoplasmic lectins has been identified in plants, possibly complementing the classical vacuolar lectins in the defense of plants against herbivores [49]. Since the cytoplasmic localization is a hallmark of fruiting body lectins, we are currently testing a whole series of such lectins for toxicity towards various organisms including *C. elegans* to provide additional experimental support for a role of these fungal lectins in defense. A recent survey of insecticidal activities in fruiting bodies suggests that most of these activities are protein-based and, thus, that the protein-mediated defense may be at least as significant for fungal physiology and ecology as the more established chemical defense of fungi by secondary metabolites [50],[51],[52].

The identified ligand of CGL2 in *C. elegans*, Galβ1,4Fucα1,6 modification of the proximal GlcNAc of N-glycan cores, was originally discovered as a feature of octopus and squid rhodopsin [53],[54] and keyhole limpet hemocyanin [55]. The physiological significance of this N-glycan modification is unknown and the identification of a novel, putative glycosyltransferase required for this modification may shed light both on its distribution and function. The biochemical characterization of the relevant *C. elegans* enzyme is published elsewhere [42]. None of the mutations affected in the biosynthesis of this specific glycoepitope showed obvious defects in development under standard laboratory conditions. In the light of the fact that these mutations were identified, because they conferred resistance towards CGL2-expressing *E. coli*, it is noteworthy that mutations in *gly-12*, *gly-13* and *gly-14*, coding for the three GnTI isoenzymes of *C. elegans*, were previously reported to affect the response of *C. elegans* to pathogenic bacteria [39]. This could suggest that the Galβ1,4Fucα1,6GlcNAc epitope, or some other GnTI-dependent modification, plays a specific role in the defense of *C. elegans* against bacterial pathogens. The fact that, of these three mutations, only *gly-13* led to resistance against CGL2-expressing *E. coli* may reflect the ubiquitous expression of this gene and the function of its product as the major GnTI in *C. elegans* [36]. On the other hand, even though the *gly-14* gene was reported to be specifically expressed in the intestine (i.e., the same tissue which we observe expresses CGL2 epitopes), the lack of resistance shown by the *gly-14* mutant can be explained by the ‘overruling’, ubiquitous expression of *gly-13* [56]. As noted above, core α1,6-fucosylation by the FUT-8 enzyme, required as the basis for subsequent galactosylation by GALT-1, the *M03F8.4* gene product, is itself dependent on the prior action of GnTI. On the other hand, GlcNAc transferases II and V (encoded by the *gly-20* and *gly-2* genes, respectively) are not prerequisites for the action of FUT-8 and, thus, the lack of resistance of *gly-2* and *gly-20* mutants to CGL2 is not surprising. The other tested fucosyltransferase mutants (*fut-1* through to *fut-6*) affect other forms of fucosylation and are not involved in the formation of the Galβ1,4Fucα1,6 epitope.

Analysis of the *C. elegans* glycome suggests that there are, besides the Galβ1,4Fucα1,6GlcNAc epitope, additional β-galactosides on N-glycans [37], on O-glycans [57] and on glycosphingolipids [5] representing potential CGL2-ligands based on the carbohydrate-binding specificity of this galectin [27]. However, the exclusive and almost complete resistance of *C. elegans* mutants affected in the biogenesis of the β1,4-galactoside on the α1,6-bound core fucose of N-glycans and the almost full sensitivity of all other tested mutants suggest that these other potential ligands are either not accessible for CGL2, not bound by CGL2 or their binding by CGL2 does not lead to toxicity. From the experiments with *B. thuringiensis* Cry5B toxin [58] and our own experiments with Thomsen-Friedenreich antigen (Galβ1,3GalNAc)-binding fungal lectins (S. Bleuler, unpublished), it can be concluded that at least some of these additional β-galactoside-containing glycoconjugates are present in the *C. elegans* intestine, making the first possibility unlikely. Since mutations in the GDP-fucose biosynthetic pathway still allowed formation of residual β-galactoside-containing glycosphingolipids [59] but led to complete loss of CGL2-binding to the intestinal epithelium (data not shown), we conclude that CGL2 does not bind these β-galactosides *in vivo*, at least not in sufficient degree to be visualized. This explanation may also apply for Thomsen-Friedenreich antigen, an epitope which is associated with *C. elegans* O-glycans and was shown to bind CGL2 *in vitro*, albeit with low affinity [28],[47],[57].

On the other hand, the sensitivity of the *bre*-mutants, except for *bre-1*, towards CGL2, suggests that the resistance of these mutants towards the *B. thuringi*ensis crystal toxin Cry5B is highly specific and that such resistances depend on the carbohydrate-binding specificity of the respective lectin/toxin. This conclusion is further validated by the sensitivity of some of the reported CGL2-resistant mutants towards other nematoxic fruiting body lectins (S. Bleuler, unpublished).

Analogous to our own results, the Galβ1,4Fucα1,6GlcNAc epitope was recently identified as a ligand for the endogenous *C. elegans* galectin LEC-6 *in vitro* [60]. It is not known, however, whether this interaction occurs also *in vivo* and what the consequences of such an interaction would be. Similar to mutations interfering with the formation of the glycoepitope (see above), knockdown of *lec-6* mRNA did not result in any obvious phenotype in *C. elegans* high-throughput functional studies [61]. In the light of the proposed role of the Galβ1,4Fucα1,6GlcNAc epitope in the response of *C. elegans* to pathogenic bacteria, it is noteworthy that expression of the *lec-6* gene was induced upon exposure of *C. elegans* to *Photorhabdus luminescens* [62].

The molecular details of the interaction between CGL2 and Galβ1,4Fucα1,6GlcNAc and other characterized ligands suggest that CGL2, and possibly many other galectins, have a relaxed specificity *in vitro* with regard to the type of linkage and the type of sugar attached to the galactose as long as the 3′ (in glucose and fucose) or 4′ (in GalNAc) OH of the linked monosaccharide is in the right orientation to interact with the conserved carbohydrate-coordinating residues, Arg55 and Glu75; of the other residues involved in binding, Arg77 only makes contact with Glc in lactose and fucose in Galβ1,4Fucα1,6GlcNAc but not with GalNAc in Thomsen-Friedenreich antigen. Our isothermal titration microcalorimetry (ITC) data does not allow conclusive statements about the relative affinity of CGL2 to Galβ1,4Fucα1,6GlcNAc vs. other non-substituted, terminal β-galactosides but taking the structural data into account, it can be estimated that the affinity to Galβ1,4Fuc is in the same range or even slightly higher than to other ligands. In an *in vivo* context, the CGL2 epitope is part of a larger N-glycan structure, which may indeed have a higher affinity towards the lectin than the trisaccharide tested here. Analogously, the role of an entire structure in optimal binding to N-glycans was shown by studies on anti-HRP which recognises core α1,3-fucosylated determinants [63]. Such a binding mode would help to explain why there is apparently no biological significance to the *in vitro* binding of CGL2 to the Thomsen-Friedenreich antigen (see discussion above).

Apart from the identity of the target glycoconjugate, we can only speculate about the mechanism of CGL2-mediated nematotoxicity: Based on the *in situ* localization of the glycoconjugate using fluorescently labeled CGL2, we assume that the recognized N-glycan is bound to one or several proteins on the epithelial membrane or the peritrophic membrane of the intestine. The fact that we isolated in our screen exclusively mutations in carbohydrate-active enzymes, suggests that toxicity is not based on the interaction between CGL2 and a particular “receptor” N-glycoprotein but is rather caused by binding of CGL2 to different N-glycoproteins characterized by the Galβ1,4Fucα1,6 epitope. A recent proteomic approach identified a number of such glycoproteins based on recognition by LEC-6 [64]. The caveats of such a conclusion are that our results are still compatible with a specific “receptor” N-glycoprotein for CGL2 and that the screen is probably not saturated and, by design, not able to identify mutations in genes that are essential for worm viability.

The intestinal localization of the CGL2-ligand in *C. elegans* coincides with the morphological changes of the intestinal epithelium upon feeding with CGL2-expressing *E. coli*. Similar morphological changes were reported in *C. elegans* in response to feeding with *E. coli* expressing the *B. thuringiensis* crystal toxin Cry5B [29] and other toxin-producing, gram-positive and gram-negative bacteria [65],[66]. Enlargement of the intestinal lumen and damage of microvillar structure of the epithelium was also observed in insect larvae upon feeding with the entomotoxic mammalian galectin-1 and snow drop lectin (GNL), respectively [67],[68]. The morphological changes are usually more pronounced in the anterior part of the intestine possibly because this part receives the highest concentration of the lectins/toxins upon feeding.

Preliminary experiments with purified CGL2 and HeLa cells suggest that the lectin is cytotoxic, rapidly endocytosed and ends up in a perinuclear compartment (M. Garbani, unpublished). This is in contrast to the *A. aegerita* galectin (AAL) which is also toxic for HeLa cells but enters the cytoplasm and nucleoplasm of the cells [48]. This difference in behavior between two different fungal galectins might be due to differences in oligomerization: AAL is a dimer whereas CGL2 forms a tetramer in solution, which might restrict its access to the cyto- and nucleoplasm [15],[28]. The cytotoxicity of AAL was recently shown to be dependent on a conserved hydrophobic patch on the protein surface in addition to domains involved in oligomerization and carbohydrate-binding [15]. At this point, we do not know whether this patch is also required for CGL2-mediated toxicity. Cytotoxicity towards mammalian cells is shared between fungal and mammalian galectins. However, ligands and toxicity mechanisms appear to vary between the different mammalian galectins and cell types [69],[70],[71],[72],[73],[74]. It remains to be tested, whether CGL2 uses one of the ligands and toxicity mechanisms identified for the mammalian galectins, whether endocytosis is necessary for cytotoxicity and how these processes relate to the nematotoxicity of CGL2.

## Materials and Methods

### Strains and cultivation conditions

*Escherichia coli* strains DH5α and BL21(DE3) were used for cloning and amplification of plasmids and bacterial expression of proteins, respectively. *E. coli* was cultivated on standard media as described [75]. *Caenorhabditis elegans* strains were maintained on nematode growth media (NGM) and fed with *E. coli* strain OP50 as described [76]. The Bristol isolate N2 was used as the wild type strain. Strains *pmk-1(km25), sek-1(ag1), nsy-1(ag3), bre-1(ye4), bre-2(ye31), bre-3(ye26), bre-4(ye27), bre-5(ye17), fut-1(ok892), fut-2(gk360), fut-2(ok509), fut-3(gk103), fut-4(gk111), fut-5(ok242), fut-6(ok475), fut-8(ok2558), gly-2(qa703), gly-12(is47), dpy-6(e14);gly-13(ok712), gly-14(id48), gly-20(ok826), unc-119(ed3)* were obtained from the *Caenorhabditis* Genetics Center (CGC) at the University of Minnesota (USA). The strain carrying the two extrachromosomal constructs *frEx113*[*hsp::MosTransposase;P_col12_::DsRed*] and *oxEx229*[*Mos1;P_myo-2_::gfp*] was kindly provided by Jonathan Ewbank. For *Mos1*-mediated mutagenesis, we generated the strains *pmk-1(km25);frEx113* and *pmk-1(km25);oxEx229.* Strains resulting from *Mos1*-mediated mutagenesis and subsequent outcrossing were *pmk-1(km25); M03F8.4(op497), pmk-1(km25); fut-8(op498), pmk-1(km25); ger-1(op499), pmk-1(km25);gly-13(op507)* and *pmk-1(km25);bre-1(op509).* Primer*s* used for genotyping are listed in Supplementary Table S3.

### Cloning and expression

Plasmid pET24-CGL2 for expression of authentic CGL2 in BL21(DE3) was described previously [27]. The plasmid for bacterial expression of *C. cinerea* CGL2(W72G) was constructed by amplifying the respective open reading frame from pYADE4-CGL2(W72G) [28] using the primers NdeI-CGL2N and BamHI-CGL2C [27] and ligating the resulting fragment into pET24a (Invitrogen) using the introduced restriction sites. Plasmid pET24-CGL1 for expression of authentic CGL1 in BL21(DE3) was constructed analogously using *cgl1*-specific primers and plasmid pBCG1 as template [26]. Expression of CGL2 and CGL2(W72G) in liquid culture was performed as described for CGL3 [27]. For the *C. elegans* bioassays, 300 µl of a overnight culture of the respective BL21(DE3) transformants were spread on NGM-plates containing 1 mM isopropyl-β-D-thiogalactoside (IPTG) and 50 µg/ml Kanamycin and incubated overnight at 23°C before addition of the nematodes. Lectin expression was verified by separating whole cell extracts of induced BL21(DE3)-transformants on Coomassie blue-stained SDS-polyacrylamide gels and immunoblotting using anti-CGL2 antiserum [26] (data not shown).

### Protein purification and labeling

Bacterial cell pellets were resuspended in ice-cold phosphate-buffered saline (PBS) [30 mM Na-phosphate pH 7.3, 150 mM NaCl] containing 1 mM phenylmethylsulfonyl fluoride and ruptured using a French press. Cell debris was removed in two consecutive steps of centrifugation at 12000 g for 15 min and 27000 g for 30 min. The supernatant was incubated with lactosyl-sepharose at 4°C for 1 h and CGL2 was finally eluted at room temperature in PBS containing 200 mM lactose. After size exclusion chromatography on Superose 6 10/300 GL (GE Healthcare) equilibrated in PBS, fractions containing the protein were pooled and concentrated using an Amicon Ultra-4 centrifugal filter device (Millipore) with a molecular weight cutoff of 10 kDa. Protein concentration was calculated by measuring the absorbance at 280 nm, assuming a relation of 1.25 mg/ml to 1 unit absorbance at 280 nm for a path length of 1 cm.

Conjugation of purified CGL2 to tetramethylrhodamine (TAMRA) (Molecular Probes) was performed as described [47].

### *C. elegans* toxicity assays

A plate assay was devised to examine the toxicity of authentic CGL1 and CGL2 and mutant CGL2 towards *C. elegans*. NGM plates were seeded with *E. coli* BL21(DE3) expressing either authentic CGL1 or CGL2 or mutant CGL2(W72G) as described above. As a control, plates were seeded with *E. coli* BL21(DE3) containing the vector pET24a. The plates were incubated overnight at 23°C and seeded with synchronized populations of *C. elegans* [77] for the different toxicity assays:

#### Microscopic analysis

For microscopic analysis of the toxic effect of CGL2 towards *C. elegans*, L4 animals were seeded onto the plates and examined after 24 h at 23°C.

#### Developmental assay

Quantitative data on the effect of CGL1 and CGL2 on *C. elegans* development was acquired by placing 50 to 100 newly hatched L1 larvae of the indicated genotypes on the plates. After 72 h, the fraction of animals that reached L4 stage was determined.

#### Brood size assay

The effect of CGL2 on *C. elegans* reproduction was assayed by picking individual L4 wild type hermaphrodites onto plates. Thereafter, the mothers were transferred to new plates daily until the mother either stopped producing offspring or died. The progeny of the previous plate were counted the next day. The number of progeny from the various plates were added up to give the final brood size.

#### Determination of the CGL2 median lethal dose (LD_50_)

We defined the LD_50_ as the concentration of toxin at which >50% of the animals fail to reach larval stage 4 (L4) within 96 h in liquid culture. 20 L1 staged *C. elegans* wild type worms were placed in wells containing S-medium [78], *E. coli* BL21 containing empty vector pET24a with a Kan^R^ gene as a food source, kanamycin and chloramphenicol (30 µg/ml each) and purified CGL2 protein in the concentrations indicated. After 96 h, the worms were transferred to NGM plates and the number of worms that reached L4 stage was determined.

#### Survival curves

For the comparison between CGL2-, CGL2(W72G)-expressing and empty vector control-containing bacteria (Fig. 1, panel C), 10 L4 stage wild type *C. elegans* (N2) were seeded onto plates with the respective bacterial lawns. Surviving animals were transferred each day onto a novel plate with the same type of bacterial lawn. For the hypersensitivity assay (Fig. 3, panel A), 10 L4 stage *C. elegans* of the indicated genotypes were transferred to plates with CGL2-expressing bacteria. The plates were checked for surviving animals every 24 h. A worm was considered dead when it did not react to touching with a worm pick.

### Statistical analysis

The statistical significance of the results was evaluated using appropriate tests. The results of the developmental toxicity assay were analyzed by the non-parametric Kolmogorov-Smirnov test, comparing pairwise each lectin to the vector control and each *C. elegans* mutant to the wildtype strain. The differences in the brood-size were compared, also pairwise, using a t test. For the statistical evaluation of the survival curves a Kaplan-Meyer analysis in combination with log rank tests was used. The difference in the CGL2-TAMRA staining between two different *C. elegans* strains was assessed using a chi square test of the respective numbers of animals showing specific staining of the intestinal epithelium (see Supplemental Table S1).

### Screen for CGL2-resistant *C. elegans* mutants using *Mos1* insertional mutagenesis

*Mos1* insertional mutagenesis was in principle performed as published [79]. We used the extrachromosomal arrays *frEx113*, which carries the *Mos1* transposase under the control of a heat-shock promoter, and *oxEx229*, which carries multiple copies of the substrate *Mos1* transposon. The two extrachromosomal constructs were crossed into CGL2-hypersensitive *pmk-1(km25)* worms to generate the two starting strains *pmk-1(km25);frEx113* and *pmk-1(km25);oEx229* for the screen. To generate double-array carrying animals, *pmk-1(km25);oxEx229* males were crossed to L4 *pmk-1(km25);frEx113* hermaphrodites. Progeny containing both arrays, recognized by the concurrent expression of GFP in the pharynx (*P_myo-2_::gfp*, contained in *oxEx229*) and DsRed in the epidermis (*P_col12_::DsRed*, contained in *frEx113*), were propagated for approximately six generations before subjecting them to heat-shock. Several hundred double-transgenic animals per mutagenesis round were subjected to heat-shock for 1 h at 33°C, 1 h at 20°C, and 1 h at 33°C and then allowed to recover overnight at 20°C. P_0_ were distributed to 90 mm NGM plates and eggs were collected 12–40 h after heat shock. P_0_ were then removed. After 3 days, gravid F_1_ were washed off the plates in M9 buffer and F_2_ eggs were isolated as described [76]. The synchronized population of F_2_ L1 animals was distributed on 30 plates containing CGL2-expressing *E. coli* BL21(DE3). After 3 to 7 days, these plates were screened for CGL2-resistant animals that had reached adulthood. Each plate with resistant animals was treated as an individual hit to avoid redundant *Mos1* insertions. Candidate worms were propagated on CGL2-expressing *E. coli* to confirm the resistance phenotype. Once resistance was confirmed, mutants that had lost the extrachromosomal *Mos1-*bearing array were outcrossed 2 to 6 times before assaying for the presence of *Mos1* elements and trying to locate the site of insertion. For those mutants that still contained a *Mos1* element, we determined the insertion site through inverse PCR on worm lysates as published [79].

In total, approximately 500′000 haploid genomes were screened. The transposition efficiency was meassured as 50%. In total, 14 CGL2-resistant worms were isolated. Only 5 of them, *pmk-1(km25);M03F8.4(op497), pmk-1(km25);fut-8(op498), ger-1(op499); pmk-1(km25), pmk-1(km25);gly-13(op507)* and *pmk-1(km25);bre-1(op509)*, contained the *Mos1* transposon insertion.

### Light microscopy of *C. elegans*

For general worm handling, a Leica MZ 12.5 stereomicroscope was used. To select double-array carrying worms (*pmk-1(km25);oxEx229;frEx113)*, we used a Leica MZ 16 FA stereomicroscope equipped with appropriate filtersets (DsRed and GFP filter). Pictures were taken with a Nikon Coolpix 990 digital camera.

For DIC and fluorescence microscopy, worms were placed on 2% agarose pads in M9 [75], anaesthesized with levamisole (3–5 mM) (Sigma) and mounted under a coverslip for observation using a Leica DM-RA or Zeiss Axiovert 200 microscope equipped with DIC (Nomarski) optics and standard epifluorescence with a DsRed filterset for detection of TAMRA. Pictures were taken with a Hamamatsu ORCA-ER camera. Images were false-coloured using OpenLab software.

For the *in situ* localization of the CGL2-ligand, L4 staged *C. elegans* were placed in wells containing S-medium [78], BL21(DE3) *E. coli* harbouring empty Kan^R^-vector, kanamycin and chloramphenicol at 30 µg/ml each and TAMRA-labeled CGL2 at 100 µg/ml. After 24 hrs, worms were transferred to standard OP50 plates and screened for TAMRA fluorescence 2 h thereafter.

### Electron microscopy of *C. elegans*

For electron microscopic examination, worms were prepared by a described two-step chemical fixation [80]. Fixation and slicing of the samples was kindly carried out by Garry Barmettler at the Center for Microscopy and Image Analysis (University of Zurich, Switzerland). The samples were examined using a Philips CM100 transmission electron microscope equipped with a side mounted digital camera (Gatan).

### Isolation of *C. elegans* N-glycans

*C. elegans* strains *pmk-1(km25)*, *pmk-1(km25);M03F8.4(op497)* and *pmk-1(km25);fut-8(op498)* were grown for 5 days at room temperature in liquid culture with *E. coli* OP50 and afterwards separated from bacteria and debris by 30% (w/v) sucrose gradient centrifugation [76]. *N*-glycan preparation was performed as previously published [81] by enzymatic release of glycans from partially purified glycopeptides using peptide-N-glycanase (PNGase) F and subsequently PNGase A in order to separate core α1,3-fucosylated glycans from other core fucosylated glycans. Briefly, approximately 3 g of worms (wet weight) were boiled prior to grinding. The extract was adjusted to contain 5% (v/v) formic acid and incubated with 3 mg pepsin (Sigma) at 37°C overnight. After centrifugation at 15′000 g for 15 min, the supernatant was applied to 15 ml Dowex AG WX2 equilibrated with 2% acetic acid. The glycopeptides were eluted with ammonium acetate (0.6 M, pH 6). Orcinol-positive fractions were pooled and lyophilized overnight. The samples were then desalted by application to a Sephadex G25 column and eluted with 1% acetic acid. The orcinol-positive fractions were again pooled and lyophilized. The samples were dissolved in 250 µl water. After heat treatment at 95°C for 5 min to inactivate residual pepsin, the samples were cooled prior to addition of 250 µl ammonium carbonate buffer pH 8 and 3 U PNGase F (Roche) and incubated overnight at 37°C. The samples were then acidified with 400 µl 10% acetic acid and applied to 5 ml Dowex AG WX2. The unretained free glycans were lyophilized and dried for subsequent fluorescent labeling whereas the retained Orcinol positive fractions eluting with ammonium acetate (0.6 M, pH 6) were desalted as above, dissolved in ammonium acetate buffer (50 mM, pH 5) and treated with PNGase A (0.6 mU) overnight at 37°C. Again the samples were acidified with 400 µl 10% acetic acid and applied to 5 ml Dowex AG WX2. The unretained free glycans were lyophilized and also dried for subsequent fluorescent labeling.

### Labeling and structural analysis of *C. elegans* N-glycans

Fluorescent labeling of the N-glycans was performed as previously described using 2-amino-pyridine (PA). Complete N-glycomes of either PNGase A or F released and pyridylaminated glycans were fractionated by 2D-HPLC using a Shimadzu HPLC system (consisting of a SCL-10A controller, two LC10AP pumps and a RF-10AXL fluorescence detector controlled by a personal computer using Class-VP software (V6.13SP2)) at room temperature and fluorescence detection (excitation at 310 or 320 nm, emission detected at 380 or 400 nm). The N-glycans were first fractionated on a normal phase HPLC (Tosoh TSK gel Amide-80, 4.6×250 mm, 5 µm; flow 1 ml/min, elution: 5 min isocratic 71.3% MeCN, 10 min gradient from 71.3% to 61.8% MeCN, 25 min isocratic 61.8% MeCN, 15 min 61.8% to 54.2% MeCN using ammonium formate (10 mM, pH 7) as buffer). The fractions were lyophilized and further fractionated on reversed phase HPLC (Hypersil ODS C-18; 4×250 mm, 5 µm; flow 1.5 ml/min, gradient of 0–9% MeOH over 30 min using ammonium formate (0.1 M, pH 4) as buffer). HPLC chromatograms were visualized using the opensource program PLOT (Version 0.997 by Wesemann and Thijsse). Each fraction was subjected to monoisotopic MALDI-TOF MS using a Bruker Ultraflex TOF/TOF with 2,5-dihydroxybenzoic acid as matrix. In general, all fractions with fucose containing N-glycans were subjected to MS/MS to elucidate their composition. A peptide standard mixture (Bruker) was used for external calibration. MS data were analysed using Bruker software and the mMass V2.4 software package [82].

Selected isolated N-glycans were examined for the presence of either terminal β-galactosides by treatment with *Aspergillus oryzae* β1,4-galactosidase (27 mU, 50 mM sodium citrate, pH 4.5) [83] for 2 days at 37°C or terminal α-fucosides by use of bovine kidney α-fucosidase (Sigma, 15 mU, 50 mM ammonium acetate, pH 5). Digestion products were subsequently analysed for altered structural characteristics by RP-HPLC (see above) and MALDI-TOF MS.

### Chemical synthesis of Galβ1,4Fucα1,6GlcNAcβOC_5_H_10_NH_2_

See Supplementary Figure S3 and Supplementary Text S1.

### Crystallization of CGL2 in complex with carbohydrate ligand

Crystallization conditions were screened using the PEG/Ion Screen from Hampton Research with the hanging-drop vapor-diffusion method at 18°C. The best crystals were obtained by mixing 2.5 µl of protein solution (10 mg/ml) containing 1 mM Galβ1,4Fucα1,6GlcNAcβOC_5_H_10_NH_2_ with 2.5 µl mother liquor consisting of 0.2 M magnesium acetate, 20% (w/v) PEG 3350. Drops were equilibrated against 500 µl reservoir solution. After 3 weeks, crystals were cryostabilized in mother liquor supplemented with 25% glycerol and flash frozen in liquid nitrogen.

### Data collection and structure determination

Diffraction data were collected at the Swiss Light Source, beamline X06DA (Villigen, Switzerland) at 100 K and processed with XDS, XSCALE and XDSCONV [84]. The structure was readily solved by molecular replacement with MOLREP [85], using the known CGL2 structure as a search model PDB ID 1UL9 [28]. The structure was refined anisotropically with Phenix [86] to 1.5 Å resolution and iterative model rebuilding was performed using Coot [87]. Model statistics were obtained with Procheck/Sfcheck as part of the CCP4 suite [85]. Molecular visualizations and structures illustrations were performed using PyMOL [88]. Data processing and refinement statistics are summarized in Supplementary Table S2. Coordinate and structure factors have been deposited with the PDB under code 2WKK.

## Supporting Information

Text S1 Chemical synthesis of Galβ1,4Fucα1,6GlcNAcβOC_5_H_10_NH_2_ (Numbering of compounds refers to Supplementary Figure S3).
(0.06 MB DOC)

Table S1 Statistics on CGL2-TAMRA staining (Fig. 6). Animals were scored for specific staining of the intestinal epithelium.
(0.01 MB PDF)

Table S2 Statistics on data collection and refinement.
(0.05 MB PDF)

Table S3 Primers used in this study.
(0.01 MB PDF)

Figure S1 Isothermal calorimetry titration curve of CGL2 with Galβ1,4Fucα1,6GlcNAcβOC_5_H_10_NH_2_. The raw data is shown in the upper panel. Transformation of the data using the Microcal software yields the titration curve (lower panel), from which the thermodynamic parameters were calulated.
(0.14 MB PDF)

Figure S2 Mass spectrometric analysis of the Hex_5_dHex_2_HexNAc_2_ glycan. The isolated fraction containing the Hex_5_dHex_2_HexNAc_2_ N-glycan as well as aliquots of this glycan treated with either fucosidase alone, galactosidase alone or sequentially with galactosidase and fucosidase were analysed by MALDI-TOF MS; the spectra are annotated with the *m/z* values for the [M+Na]^+^ species (left). The corresponding MALDI-TOF MS/MS fragmentation spectra are also shown and annotated with the putative structures of key diagnostic fragments (right).
(0.05 MB PDF)

Figure S3 The synthesis of Galβ1,4Fucα1,6GlcNAcβOC_5_H_10_NH_2_. Reaction conditions: a) AcOH, 50°C; b) Bu_2_SnO, toluene, reflux; c) BnBr, TBAI, 40°C; d) FmocCl, pyridine; e) K_2_CO_3_, Cl_3_CCN, DCM; f) N-(benzyl)-benzyloxycarbonyl-5-aminopentan-1-ol, TMSOTf, DCM, -15°C; g) HF-pyridine, THF; h) 4, DMTST, DTBMP, DCM, -10°C; i) Et_3_N; j) 2,3,4,6-tetra-*O*-benzoyl-*β*-D-galactopyranosyl trichloroacetimidate, TMSOTf, DCM, -10°C; k) ethylenediamine, *n*BuOH, reflux; l) Ac_2_O, pyridine; m) NaOMe, MeOH; n) Pd/C, H_2_, MeOH/H_2_O/AcOH.
(0.12 MB PDF)
