# Supplementary material for: Caenorhabditis elegans N-glycan Core β-galactoside Confers Sensitivity towards Nematotoxic Fungal Galectin CGL2
Source: PLoS Pathog. 2010 Jan 8;6(1):e1000717. doi: 10.1371/journal.ppat.1000717 (PMC2798750; doi:10.1371/journal.ppat.1000717)
Supplement: Text S1 — Chemical synthesis of Galβ1,4Fucα1,6GlcNAcβOC5H10NH2 (Numbering of compounds refers to Supplementary Figure S3). (0.06 MB DOC) [file ppat.1000717.s001.doc]

**Supporting Information: Supplementary Text**

**Chemical synthesis of Galβ1,4Fucα1,6GlcNAcβOC_5_H_10_NH_2_ (Numbering of compounds refers to Supplementary Figure S3)**

**Phenyl 2-*O*-benzyl-1-thio-*β*-L-fucopyranoside (2)**

2.15 g (5.56 mmol) thio fucoside **1** was dissolved in 75% aqueous AcOH (40 mL). The reaction mixture was stirred at 50 °C for 1 h, then concentrated. The residue was diluted with DCM and washed with saturated NaHCO_3_ and brine, dried over MgSO_4_, filtered and concentrated. Flash column chromatography on silica gel (hexane/EtOAc 1:1) afforded **2** (1.78 g, 92%) as a colorless oil. ^1^H NMR (400 MHz, CDCl_3_) δ 7.60-7.58 (m, 2H), 7.43-7.31 (m, 8H), 4.86 (ABq, *J* = 11.0 Hz, *CH_2_*Ph, 2H), 4.63 (d, *J* = 9.6 Hz, *H*-1, 1H), 3.73 (m, *H*-3, 1H), 3.66 (m, *H*-4, 1H), 3.61 (td, *J* = 6.3, 0.81 Hz, *H*-5, 1H), 3.58 (dd, *J* = 9.6, 9.0 Hz, *H*-2, 1H), 2.80 (d, *J* = 5.2 Hz, *OH*-4, 1H), 2.47 (d, *J* = 5.2 Hz, *OH*-3, 1H), 1.36 (d, *J* = 6.5 Hz, *CH_3_*-6, 3H). ^13^C NMR (101 MHz, CDCl_3_) δ 138.17, 134.11, 131.68, 128.95, 128.59, 128.46, 128.28, 128.06, 127.44, 87.47, 78.14, 77.41, 77.29, 77.09, 76.77, 75.32, 75.31, 74.47, 71.78, 16.65. IR (neat): 3403, 3061, 3031, 2982, 2868, 1583, 1497, 1480, 1454, 1440, 1399, 1382, 1365, 1276, 1214, 1159, 1073, 1048, 1028, 995, 941, 913, 889, 862, 801, 736, 692, 667, 612 cm^-1^. [α]_D_ = -12.5 (*c* = 1.0, CHCl_3_). MALDI-HRMS: *m/z* calcd. for C_19_H_22_O_4_SNa [M+Na]^+^ 369.1131, obsd. 369.1135.

**Phenyl 2,3-di-*O*-benzyl-1-thio-*β*-L-fucopyranoside (3)**

To a solution of **2** (1.135g, 3.28 mmol) in toluene (40 mL), Bu_2_SnO (890 mg, 3.60 mmol) was added. The mixture was heated to reflux for 3 h. During the reflux, water was removed continuously, until the homogeneous solution formed. The solution was then concentrated to about 10 mL, then BnBr (1 mL, 8.36 mmol) and TBAI (600 mg, 1.64 mmol) were added. The reaction mixture was stirred at room temperature overnight, and concentrated. Flash column chromatography on silica gel (hexane/EtOAc 5:1) afforded **3** (1.31 g, 92%) as a colorless oil. ^1^H NMR (400 MHz, CDCl_3_) δ 7.50-7.48 (m, 2H), 7.33-7.18 (m, 13H), 4.71 (ABqd, *J* = 10.3, 1.67 Hz, *CH_2_*Ph, 2H), 4.62 (m, *CH_2_*Ph, 2H), 4.52 (dd, *J* = 9.7, 2.28 Hz, *H*-1, 1H), 3.73 (m, *H*-4, 1H), 3.61 (ddd, *J* = 9.6, 9.6, 2.14 Hz, *H*-2, 1H), 3.49 (m, *H*-3 and *H*-5, 2H), 2.23 (br s, *OH*-4, 1H), 1.29 (dd, *J* = 6.4, 2.2 Hz, *CH_3_*-6, 3H). ^13^C NMR (101 MHz, CDCl_3_) δ 138.31, 137.75, 134.04, 131.97, 128.87, 128.58, 128.38, 128.28, 128.04, 127.92, 127.81, 127.38, 87.59, 82.95, 77.40, 77.29, 77.08, 76.91, 76.77, 75.71, 74.25, 72.18, 69.42, 16.79. IR (neat): 3423, 2981, 2890, 1583, 1497, 1476, 1454, 1438, 1408, 1377, 1362, 1280, 1265, 1219, 1206, 1165, 1123, 1099, 1078, 1056, 1026, 1008, 994, 979, 948, 908, 860, 805, 751, 734, 700, 690, 670, 620 cm^-1^. [α]_D_ = -1.51 (*c* = 1.0, CHCl_3_). MALDI-HRMS: *m/z* calcd. for C_26_H_28_O_4_SNa [M+Na]^+^ 459.1601, obsd. 459.1601.

**Phenyl 2,3-di-*O*-benzyl-4-*O*-fluorenylmethoxy-carbonyl-1-thio-*β*-L-fucopyranoside (4)**

To a solution of **3** (323 mg, 0.74 mmol) in pyridine (7 mL), FmocCl (294.4 mg, 1.14 mmol) was added. The reaction mixture was stirred at room temperature for 3 h and then concentrated. Flash column chromatography on silica gel (hexane/EtOAc 10:1) afforded **4** (418 mg, 85%) as a colorless oil. ^1^H NMR (400 MHz, CDCl_3_) δ 7.83 (d, *J* = 7.6 Hz, 2H), 7.73-7.66 (m, 4H), 7.49-7.23 (m, 17H), 5.24 (d, *J* = 3.1 Hz, *H*-4, 1H), 4.83 (ABq, *J* = 10.3 Hz, *CH_2_*Ph, 2H), 4.71 (ABq, *J* = 11.4 Hz, *CH_2_*Ph, 2H), 4.70 (d, *J* = 9.3 Hz, *H*-1, 1H), 4.48 (ABqd, *J* = 10.4, 7.4 Hz, *CH_2_*, 2H), 4.31 (t, *J* = 7.4 Hz, *CH*, 1H), 3.83 (dd, *J* = 9.3, 9.3 Hz, *H-*2, 1H), 3.76 (td, *J* = 6.5, 0.7 Hz, *H-*5, 1H), 3.72 (dd, *J* = 9.1, 3.2 Hz, *H*-3, 1H), 1.37 (d, *J* = 6.4 Hz, *CH_3_-6*, 3H). ^13^C NMR (101 MHz, CDCl_3_) δ 155.49, 143.70, 143.23, 141.40, 141.32, 138.32, 137.67, 133.80, 131.96, 128.90, 128.38, 128.33, 128.29, 127.94, 127.93, 127.88, 127.81, 127.76, 127.45, 127.20, 125.41, 125.22, 120.07, 87.52, 81.23, 76.76, 76.48, 75.79, 74.25, 72.91, 72.00, 70.03, 46.78, 16.79. IR (neat): 3032, 2864, 1740, 1584, 1497, 1479, 1452, 1388, 1365, 1260, 1235, 1152, 1109, 1088, 1058, 1026, 975, 955, 904, 873, 807, 780, 737, 696 cm^-1^. [α]_D_ = -5.9 (*c* = 1.0, CHCl_3_). MALDI-HRMS: *m/z* calcd. for C_41_H_38_O_6_SNa [M+Na]^+^ 681.2281, obsd. 681.2291.

**2-*N*-Phthalimido-3,4-di-*O*-acetyl-6-*O*-*tert*-butyldiphenylsilyl-2-deoxy-*β*-D-glucopyranosyl trichloroacetimidate (6)**

To a solution of **5** (103.9 mg, 0.164 mmol) in DCM (1.5 mL), K_2_CO_3_ (114 mg, 0.825 mmol) and Cl_3_CCN (164 µL, 1.64 mmol) were added. The reaction mixture was stirred at room temperature overnight and then concentrated. Flash column chromatography on silica gel (hexane/EtOAc 3:1) afforded **6** (117.9 mg, 92%) as a colorless oil. ^1^H NMR (400 MHz, CDCl_3_) δ 8.58 (s, *NH*, 1H), 7.75 (m, 2H), 7.64-7.62 (m, 6H), 7.29-7.27 (m, 6H), 6.61 (d, *J* = 8.7 Hz, *H*-1, 1H), 5.86 (dd, *J* = 10.7, 9.1 Hz, *H*-3, 1H), 5.32 (dd, *J* = 9.3, 9.9 Hz, *H*-4, 1H), 4.56 (dd, *J* = 10.7, 8.8 Hz, *H*-2, 1H), 3.87 (ddd, *J* = 10.1, 4.2, 2.0 Hz, *H*-5, 1H), 3.79 (ABqd, *J* = 11.7, 2.0 Hz, *H*-6, 1H), 3.72 (ABqd, *J* = 11.7, 4.3 Hz, *H*-6’, 1H), 1.95 (s, *Ac*, 3H), 1.88 (s, *Ac*, 3H), 0.98 (s, *tBu*, 9H). ^13^C NMR (101 MHz, CDCl_3_) δ 171.05, 170.21, 169.19, 167.44, 160.45, 135.76, 135.68, 134.39, 133.22, 133.15, 131.30, 129.70, 129.66, 127.84, 127.68, 127.63, 123.65, 93.43, 90.37, 75.32, 70.90, 68.75, 62.25, 60.34, 53.82, 26.69, 21.01, 20.60, 20.49, 19.29, 14.20. MALDI-HRMS: *m/z* calcd. for C_36_H_37_Cl_3_N_2_O_9_SiNa [M+Na]^+^ 797.1232, obsd. 797.1237.

***N*-(Benzyl)-benzyloxycarbonyl-5-aminopentyl 2-*N*-phthalimido-3,4-di-*O*-acetyl-6-*O*-*tert*-butyldiphenylsilyl-2-deoxy-*β*-D-glucopyranoside (7)**

Glucosamine trichloroimidate **6** (117.9 mg, 0.152 mmol) and the linker N-(benzyl)-benzyloxycarbonyl-5-aminopentan-1-ol (49.3 mg, 0.151 mmol) were coevaporated three times with toluene, dried *in vacuo* overnight, and dissolved in DCM (0.6 mL). At -15 °C, TMSOTf (7 µL, 0.038 mmol) was added. The reaction mixture was slowly warmed to room temperature over 1 h, quenched with pyridine (0.1 mL), and concentrated. The crude compound **7** was directly used in the next step without further purification.

***N*-(Benzyl)-benzyloxycarbonyl-5-aminopentyl 2-*N*-phthalimido-3,4-di-*O*-acetyl-2-deoxy-*β*-D-glucopyranoside (8)**

HF-pyridine (0.13 mL, 1.46 mmol) was added dropwise to a solution of **7** (137.3 mg, 0.146 mmol) in THF (1.4 mL). The reaction mixture was stirred at room temperature overnight, quenched with saturated NaHCO_3_ and diluted with DCM. The aqueous phase was washed three times with DCM, the organic phases were combined, washed with saturated NaHCO_3_ and brine, dried over MgSO_4_, then filtered and concentrated. Flash column chromatography on silica gel (hexane/EtOAc 1:1) afforded **8** (100.3 mg, 98%) as a colorless oil. ^1^H NMR (400 MHz, CDCl_3_) δ 7.70 (m, 2H), 7.56 (m, 2H), 7.24-7.18 (m, 8H), 7.10-7.05 (m, 2H), 5.74 (dd, *J* = 10.8, 9.1 Hz, *H*-3, 1H), 5.29 (d, *J* = 7.0 Hz, *H*-1, 1H), 5.06 (s, *CH_2_*Ph, 2H), 5.03 (dd, *J* = 9.4 Hz, *H*-4, 1H), 4.31 (s, *CH_2_*Ph, 2H), 4.21 (dd, *J* =10.8, 8.5 Hz, *H*-2, 1H), 3.72-3.60 (m, 4H), 3.34-3.29 (m, 2H), 2.93-2.83 (m, 2H), 1.98 (s, *Ac*, 3H), 1.79 (s, *Ac*, 3H), 1.38-1.12 (m, 6H), 1.21-0.91 (m, 2H). ^13^C NMR (101 MHz, CDCl_3_) δ 170.18, 137.89, 134.27, 131.38, 128.52, 128.44, 127.93, 127.82, 127.27, 127.15, 123.53, 98.10, 74.17, 70.79, 69.71, 69.48, 67.14, 61.43, 54.78, 29.69, 28.87, 22.89, 20.67, 20.46. IR (neat): 3474, 2945, 2336, 2179, 1751, 1717, 1469, 1424, 1388, 1234, 1076, 1037, 910, 723, 700 cm^-1^. [α]_D_ = 12.6 (*c* = 1.0, CHCl_3_). MALDI-HRMS: *m/z* calcd. for C_38_H_42_N_2_O_11_Na [M+Na]^+^ 725.2681, obsd. 725.2694.

***N*-(Benzyl)-benzyloxycarbonyl-5-aminopentyl (2,3-di-*O*-benzyl-*α*-L-fucopyranosyl)-(1→6)-2-*N*-phthalimido-3,4-di-*O*-acetyl-2-deoxy-*β*-D-glucopyranoside (9)**

DMTST was made freshly: MeSSMe (46 µL, 0.51 mmol) and MeOTf (58 µL, 0.51 mmol) were mixed together. About 5 min solid was formed, and then dissolved in anhydrous DCM (1 mL). Fucose thioglycoside **4** (56 mg, 0.085 mmol) and glucosamine **8** (48.8 mg, 0.074 mmol) were coevaporated three times with toluene, dried *in vacuo* overnight, and dissolved in DCM (2 mL). At -10 °C, DMTST was added. The reaction mixture was slowly warmed to room temperature over 1 h, quenched with Et_3_N (2 mL), stirred for 1 h and concentrated. Flash column chromatography on silica gel (hexane/EtOAc 1:1) afforded disaccharide **9** (54 mg, 75%) as a colorless oil. ^1^H NMR (400 MHz, CDCl_3_) δ 7.69 (m, 2H), 7.55 (m, 2H), 7.29-7.18 (m, 20H), 5.70 (dd, *J* = 10.8, 9.0 Hz, *H*-3, 1H), 5.24 (d, *J* = 8.5 Hz, *H*-1, 1H), 5.04 (s, 2H), 5.01 (dd, *J* = 10.0, 9.1 Hz, *H*-4, 1H), 4.82 (d, *J* = 3.5 Hz, *H*-1’, 1H), 4.74-4.56 (m, 4H), 4.28 (s, 2H), 4.20 (dd, *J* = 10.8, 8.5 Hz, *H*-2, 2H), 3.88 (dd, *J* = 13.4, 6.8 Hz, 1H), 3.80 (m, *H*-5, 1H), 3.77 (m, 2H), 3.75 (dd, *J* = 9.8, 3.5 Hz, *H-*2’, 1H), 3.69-3.60 (m, *H*-3’ and *H*-4’, 2H), 3.22 (m, 1H), 2.82 (m, 2H), 1.90 (s, *Ac*, 3H), 1.78 (s, *Ac*, 3H), 1.20 (d, *J* = 6.6 Hz, *CH_3_-*6’, 3H), 1.25-1.14 (m, 6H), 0.88 (m, 2H). ^13^C NMR (101 MHz, CDCl_3_) δ 170.22, 169.61, 156.58, 138.66, 138.29, 137.91, 134.24, 131.40, 128.51, 128.47, 128.37, 127.81, 127.66, 127.63, 127.26, 123.51, 97.85, 77.67, 77.35, 77.23, 77.03, 76.71, 75.79, 73.41, 72.94, 72.70, 71.00, 70.20, 70.07, 69.47, 67.09, 66.97, 65.49, 60.37, 54.68, 50.42, 46.92, 28.93, 23.05, 21.02, 20.70, 20.48, 16.18, 14.20. MALDI-HRMS: *m/z* calcd. for C_58_H_64_N_2_O_15_Na [M+Na]^+^ 1051.420, obsd. 1051.431.

***N*-(Benzyl)-benzyloxycarbonyl-5-aminopentyl (2,3,4,6-tetra-*O*-benzoyl-*β*-D-galactopyranosyl)-(1→4)-(2,3-di-*O*-benzyl-*α*-L-fucopyranosyl)-(1→6)-2-*N*-phthalimido-3,4-di-*O*-acetyl-2-deoxy-*β*-D-glucopyranoside (10)**

Disaccharide **9** (29.6 mg, 0.029 mmol) and 2,3,4,6-tetra-*O*-benzoyl-*β*-D-galactopyranosyl trichloroacetimidate (32.0 mg, 0.432 mmol) were coevaporated three times with toluene, dried *in vacuo* overnight, and dissolved in DCM (0.6 mL). At -10 °C, TMSOTf (7 mL, 0.038 mmol) was added. The reaction mixture was slowly warmed to room temperature over 1 h, quenched with Et_3_N (0.1 mL), and concentrated. Flash column chromatography on silica gel (hexane/EtOAc 1:1) afforded trisaccharide **10** (44 mg, 94%) as a white foam. ^1^H NMR (400 MHz, CDCl_3_) δ 8.04-7.68 (m, 14H), 7.27-7.15 (m, 30H), 5.82 (d, *J* = 3.0 Hz, 1H), 5.77 (dd, *J* = 10.2, 8.4 Hz, *H*-2’’, 1H), 5.70-5.65 (m, 2H), 5.49-5.39 (m, 2H), 5.41 (dd, *J* = 10.4, 3.5 Hz, *H*-3’’, 1H), 5.21-5.18 (m, 1H), 5.19 (d, *J* = 8.4 Hz, *H*-1, 1H), 5.04-4.99 (m, 4H), 4.98 (d, *J* = 7.8 Hz, *H*-1’’, 1H), 4.96 (m, *H*-4, 1H), 4.93-4.87 (m, 1H), 4.78 (m, *H*-3’, 1H), 4.77 (d, *J* = 3.6 Hz, *H*-1’, 1H), 4.78-4.70 (m, *CH_2_*Ph and *H*-2’, 3H), 4.59-4.56 (m, 2H), 4,35-4.27 (m 4H), 4.23-4.13 (m, *CH_2_*Ph and *H*-2, 3H), 4.08-3.94 (m, *CH_2_*Ph and *H*-4’ and *H*5’, 4H), 3.94-3.81 (m, 6H), 3.17 (br, 1H), 2.76 (m, 2H), 1.87 (s, *Ac*, 3H),1.78 (s, *Ac*, 3H), 1.21 (d, *J* = 6.9 Hz, *CH_3_-*6’, 3H), 1.25-1.14 (m, 6H), 0.84 (m, 2H). ^13^C NMR (101 MHz, CDCl_3_) δ 171.14, 170.21, 169.61, 165.65, 165.05, 138.85, 138.76, 137.92, 136.76, 134.23, 133.44, 133.16, 131.38, 130.10, 129.97, 129.92, 129.78, 129.73, 129.57, 129.48, 129.18, 128.85, 128.56, 128.51, 128.42, 128.34, 128.28, 127.81, 127.74, 127.64, 127.50, 127.26, 123.50, 102.06, 98.11, 97.83, 79.16, 77.90, 77.35, 77.03, 76.71, 76.03, 73.22, 72.18, 71.44, 71.00, 70.04, 69.45, 68.22, 67.08, 66.20, 61.79, 60.37, 54.68, 29.69, 28.86, 23.02, 21.03, 20.70, 20.48, 16.82, 16.25, 14.20. MALDI-HRMS: *m/z* calcd. for C_92_H_90_N_2_O_24_Na [M+Na]^+^ 1629.578, obsd. 1629.584.

***N*-(Benzyl)-benzyloxycarbonyl-5-aminopentyl (2,3,4,6-tetra-*O*-acetyl-*β*-D-galactopyranosyl)-(1→4)-(2,3-di-*O*-benzyl-*α*-L-fucopyranosyl)-(1→6)-2-*N*-acetamido-3,4-di-*O*-acetyl-2-deoxy-*β*-D-glucopyranoside (11)**

Ethylenediamine (6 mL) was added to a solution of trisaccharide **10** (35.4 mg, 0.022 mmol) in BuOH (6 mL). The reaction mixture was stirred under refluxing for 6 h, concentrated and coevaporated twice with toluene. The residue was dissolved in pyridine (3 mL), then Ac_2_O (0.5 mL) was added. The reaction mixture was stirred at room temperature overnight, quenched by adding MeOH at 0 °C, then concentrated. Flash column chromatography on silica gel (hexane/EtOAc 1:2 → 1:4) afforded **11** (20.4 mg, 73%) as a white foam.

**5-Aminopentyl (*β*-D-galactopyranosyl)-(1→4)-(*α*-L-fucopyranosyl)-(1→6)-2-*N*-acetamido-2-deoxy-*β*-D-glucopyranoside (Gal*β*1,4Fuc*α*1,6GlcNAcβOC_5_H_10_NH_2_, 12)**

To a solution of trisaccharide **11** (20.4 mg) in MeOH (0.5 mL), NaOMe (0.5 M in MeOH, 0.2 mL) was added. The reaction mixture was stirred overnight, then neutralized by adding Amberlite 120, and finally concentrated. The residue was then dissolved in MeOH/H_2_O 5:1 (5 mL). Then two drops of AcOH and 10% Pd/C (50 mg) was added and H_2_ was bubbled through for 10 min. The reaction mixture was stirred under H_2_ atmosphere at room temperature for two days. Pd/C was then filtered, the solution was concentrated. The residue was purified by passing Sexphadex G-25 (MeOH/H_2_O 1:10), and lyophilized to afford **12** (9.2 mg, 93%) as a white foam. ^1^H NMR (400 MHz, D_2_O) δ 4.94 (d, *J* = 3.7 Hz, *H*-1’, 1H), 4.47 (d, *J* = 8.4 Hz, *H*-1, 1H), 4.41 (d, *J* = 7.1 Hz, *H*-1’’, 1H), 4.18 (td, *J* = 6.7, 5.7 Hz, *H*-5’, 1H) 4.02 (dd, *J* = 8.2, 5.5 Hz, *H*-4’, 1H), 3.97-3.94 (m, *H*-3’, 1H), 3.88 (dd, *J* = 6.4, 3.2 Hz, 1H), 3.85-3.81 (m, 1H), 3.79-3.77 (dd, *J* = 3.9, 1.8 Hz, 1H), 3.75 (dd, *J* = 6.2, 3.8 Hz, *H*-2’, 1H), 3.73-3.71 (m, 1H), 3.69-3.66 (dd, *J* = 7.1, 4.6 Hz, 1H), 3.65-3.63 (m, 1H), 3.61-3.60 (dd, *J* = 3.1, 2.7 Hz, 1H), 3.58-3.52 (m, 2H), 3.50-3.42 (m, 2H), 2.94 (dd, *J* = 7.8, 7.5 Hz, 2H), 1.66-1.61 (m, 2H), 1.56-1.52 (m, 2H), 1.28 (d, *J* = 6.5 Hz, *CH_3_-*6’, 3H), 1.39-1.25 (m, 2H). ^13^C NMR (101 MHz, D_2_O) δ 174.49, 132.84, 108.75, 103.49, 101.24, 99.34, 82.44, 81.80, 80.87, 80.39, 79.95, 76.04, 75.24, 75.01, 73.72, 72.57, 71.38, 70.42, 70.10, 69.94, 68.95, 68.84, 68.67, 67.43, 67.05, 66.76, 62.77, 61.12, 55.62, 48.94, 42.63, 39.37, 32.65, 28.16, 26.43, 25.07, 23.31, 22.19, 16.29, 15.34. MALDI-HRMS: *m/z* calcd. for C_25_H_46_N_2_O_15_Na [M+Na]^+^ 637.2790, obsd. 637.2794.
