# Supplementary material for: Caenorhabditis elegans N-glycan Core β-galactoside Confers Sensitivity towards Nematotoxic Fungal Galectin CGL2
Source: PLoS Pathog. 2010 Jan 8;6(1):e1000717. doi: 10.1371/journal.ppat.1000717 (PMC2798750; doi:10.1371/journal.ppat.1000717)
Supplement: Table S1 — Statistics on CGL2-TAMRA staining (Fig. 6). Animals were scored for specific staining of the intestinal epithelium. (0.01 MB PDF) [file ppat.1000717.s002.pdf]

Supporting Information: Supplementary Table S1

Table S1. Statistics on CGL2-TAMRA staining (Fig. 6). Animals were scored for specific staining of the intestinal epithelium.

Strain	***# of animals scored***	***# of animals stained***	***# of animals not stained***	***Fraction of animals stained***
pmk-1(km25)	32	31	1	0.97
fut-8(op498);pmk-1(km25)	25	4	21	0.16
