# Supplementary material for: Caenorhabditis elegans N-glycan Core β-galactoside Confers Sensitivity towards Nematotoxic Fungal Galectin CGL2
Source: PLoS Pathog. 2010 Jan 8;6(1):e1000717. doi: 10.1371/journal.ppat.1000717 (PMC2798750; doi:10.1371/journal.ppat.1000717)
Supplement: Table S2 — Statistics on data collection and refinement. (0.05 MB PDF) [file ppat.1000717.s003.pdf]

Supporting Information: Supplementary Table S2

Table S2. Statistics on Data Collection and Refinement

	**CGL2/Galβ1,4Fucα1,6GlcNAc complex (PDB ID 2wkk)**
Data Collection	
Space Group	$P2_12_12_1$
Unit cell (Å)	a=66.21; b=80.62; c=120.63
Beamline	X06DA at SLS
Wavelength (Å)	1.0
Maximum resolution (Å)	1.50
High resolution shell	1.60 – 1.50
Redundancy	12.68 (8.17)[a]
Completeness (%)	99.7 (98.3)[a]
R_{meas} (%)	7.3 (79.1)[a]
I / σ	23.08 (2.74)[a]
Refinement	
R_{cryst} (%), reflections	15.0 (103536)
R_{free} (%), reflections	18.6 (1999)
Number of atoms	
Protein atoms	4780
Water	815
Ligand	141
Average B factor ($Å^2$)	
Protein atoms	18.1
Water	34.4
Ligand	39.1
R.m.s.d. bond length (Å)	0.009
R.m.s.d. angles length (°)	1.685
Ramachandran plot [b] (%)	
Residues in core	96.5
Allowed	3.5
Generously allowed	0

[a] Values in parentheses correspond to high resolution shell in data collections

[b] Statistics from Procheck [Collaborative Computational Project, No. 4 (1994)]
