# Supplementary material for: Caenorhabditis elegans N-glycan Core β-galactoside Confers Sensitivity towards Nematotoxic Fungal Galectin CGL2
Source: PLoS Pathog. 2010 Jan 8;6(1):e1000717. doi: 10.1371/journal.ppat.1000717 (PMC2798750; doi:10.1371/journal.ppat.1000717)
Supplement: Table S3 — Primers used in this study. (0.01 MB PDF) [file ppat.1000717.s004.pdf]

Supporting Information: Supplementary Table S3

Table S3. Primers used in this study

Primer name	Nucleotide sequence	Purpose
oJL102	CAACCTTGACTGTCGAACCACCATAG	*Mos1* mutagenesis
oJL103	TCTGCGAGTTGTTTTTGCGTTTGAG	*Mos1* mutagenesis
oJL104b	ACAAAGAGCGAACGCAGACGAGT	*Mos1* mutagenesis
oJL114	AAAGATTCAGAAGGTCGGTAGATGGG	*Mos1* mutagenesis
oJL115	GCTCAATTCGCGCCAAACTATG	*Mos1* mutagenesis
oJL116	GAACGAGAGGCAGATGGAGAGG	*Mos1* mutagenesis
iPCR1a	GACCTTGTGAAGTGTCAACCTTGACTG	*Mos1* mutagenesis
iPCR1b	GACAATCGATAAATATTTACGTTTGCGAGAC	*Mos1* mutagenesis
iPCR2b	CATCTATATGTTCGAACCGACATTCCC	*Mos1* mutagenesis
fut8_mos1fw	ATCCAACTGTAGTACCAGAAGCC	genotyping *op498*
fut8mos1_rev	CAATCATTCACCACTTTATATGATGGG	genotyping *op498*
M03F4.8fw	AGCATGCTTTTGCATTCTCGGC	genotyping *op497*
M03F8_4Mos1r	ATCTTGAATTCTATACCGAATCGGC	genotyping *op497*
ger1_mos1	AACTGGCTTAGTTGGAAGCGC	genotyping *op499*
ger1mos1_rev	GACCCATTTCTCATCGTCTCGC	genotyping *op499*
gly13_Mos1_F	TTTCCACCTAAAATCATGCATGCGG	genotyping *op507*
gly13_Mos1_R	TTGTGATATTCGCTTTATCGCCGGCC	genotyping *op507*
bre-1_Mos1_F	AATTTATTGATTTTTGTGGCCCGGC	genotyping *op509*
bre-1_Mos1_R	AATGATGTCTCAATGGAGCGCAC	genotyping *op509*
bre-1_wt_fw	CCTACAATATGTTTGCTTGCAATAG	genotyping *ye4*
bre-1_ye4_fw	CCTACAATATGTTTGCTTGCAATAA	genotyping *ye4*
bre-1_rev	TCAGCATTTCCCAATAAAGTCTCC	genotyping *ye4*
pmk-1_f	TTTGTGTCCCTAATTCCTTGATCTC	genotyping *km25*
pmk-1_r	TGAGTCCACGAAGAATTTGATAGAC	genotyping *km25*
ok2558fwd	TTGGTGCGAGAAGAACACAG	genotyping *ok2558*
ok2558rev	CATCAACTCCCACCAAATCC	genotyping *ok2558*
fut-1_f	TTGCTGTTTGTCTGACAATGGC	genotyping *ok892*
fut-1_r	TTAACAGTTCTCATTTTTCTCCGC	genotyping *ok892*
hex-2_f	TGTGGATATGATTCTGAGCGAGGC	genotyping *ok1764*
hex-2_r	TGAAGTTTCTCCGACATTTCCCCG	genotyping *ok1764*
aman-2_f	GAAAAAGTTGGAAGTTGCTCGCC	genotyping *tm1078*
aman-2_r	GCATAAATTGAATATTTAGGCGCCATTCC	genotyping *tm1078*
