# Supplementary material for: Caenorhabditis elegans N-glycan Core β-galactoside Confers Sensitivity towards Nematotoxic Fungal Galectin CGL2
Source: PLoS Pathog. 2010 Jan 8;6(1):e1000717. doi: 10.1371/journal.ppat.1000717 (PMC2798750; doi:10.1371/journal.ppat.1000717)
Supplement: Figure S1 — Isothermal calorimetry titration curve of CGL2 with Galβ1,4Fucα1,6GlcNAcβOC5H10NH2. The raw data is shown in the upper panel. Transformation of the data using the Microcal software yields the titration curve (lower panel), from which the thermodynamic parameters were calulated. (0.14 MB PDF) [file ppat.1000717.s005.pdf]

Supporting Information: Supplementary Figure S1

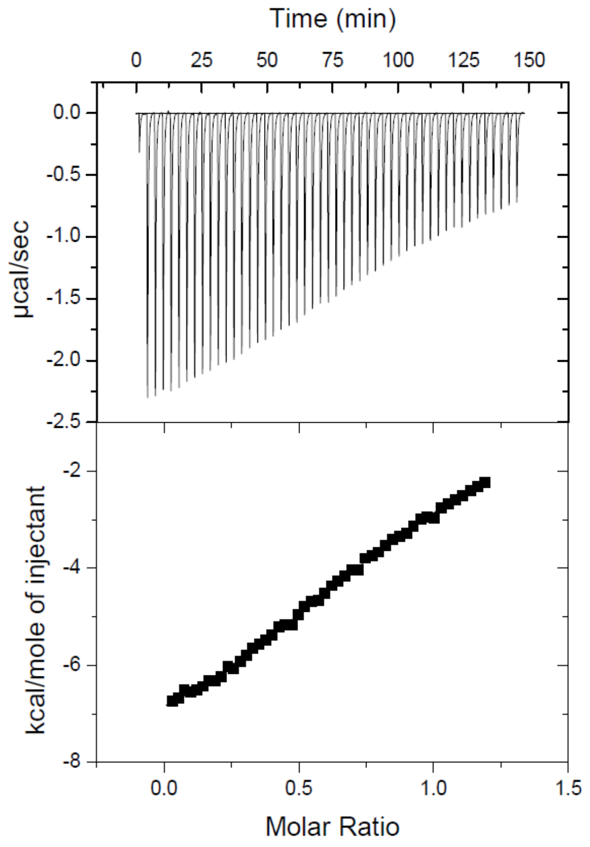

Figure S1. Isothermal calorimetry titration curve of CGL2 with Galβ1,4Fucα1,6GlcNAcβ$OC_5H_{10}NH_2$. The raw data is shown in the upper panel. Transformation of the data using the Microcal software yields the titration curve (lower panel), from which the thermodynamic parameters were calulated.

Isothermal titration microcalorimetry (ITC) measurements

Experiments were performed with a VP-ITC isothermal titration calorimeter (Microcal) at 25 °C using purified CGL2 (300 µM in TBS, purification as described in Materials and Methods). Galβ1,4Fucα1,6GlcNAcβ$OC_5H_{10}NH_2$ was dissolved in TBS at a concentration of 1.6 mM corrected based on purity estimate by ^{1}H-NMR (ca. 50-60 %). The ligand was titrated into a cell containing the protein solution in 48 injections of 6 µl preceded by a single injection of 2 µl. All injections were performed at intervals of 3 min while stirring at 270 rpm. Experimental data were fitted to a theoretical titration curve using the MCS-ORIGIN software supplied by Microcal. The number of binding sites ($N = 0.977 \pm 0.004$), the association constant ($K_a = 1.15 \pm 0.044 \times 10^4$ M^{-1}) and enthalpy change ($\Delta H = -8.85 \pm 0.1$ kcal/mol) were obtained using a model of one ligand-binding site per protein monomer. Dissociation constants ($K_d = 86.9 \pm 3.2 \times 10^{-6}$ M) and entropy of binding ($T\Delta S = -3.31$ kcal/mol) were calculated.
