# Supplementary material for: Caenorhabditis elegans N-glycan Core β-galactoside Confers Sensitivity towards Nematotoxic Fungal Galectin CGL2
Source: PLoS Pathog. 2010 Jan 8;6(1):e1000717. doi: 10.1371/journal.ppat.1000717 (PMC2798750; doi:10.1371/journal.ppat.1000717)
Supplement: Figure S2 — Mass spectrometric analysis of the Hex5dHex2HexNAc2 glycan. The isolated fraction containing the Hex5dHex2HexNAc2 N-glycan as well as aliquots of this glycan treated with either fucosidase alone, galactosidase alone or sequentially with galactosidase and fucosidase were analysed by MALDI-TOF MS; the spectra are annotated with the m/z values for the [M+Na]+ species (left). The corresponding MALDI-TOF MS/MS fragmentation spectra are also shown and annotated with the putative structures of key diagnostic fragments (right). (0.05 MB PDF) [file ppat.1000717.s006.pdf]

Supporting Information: Supplementary Figure S2

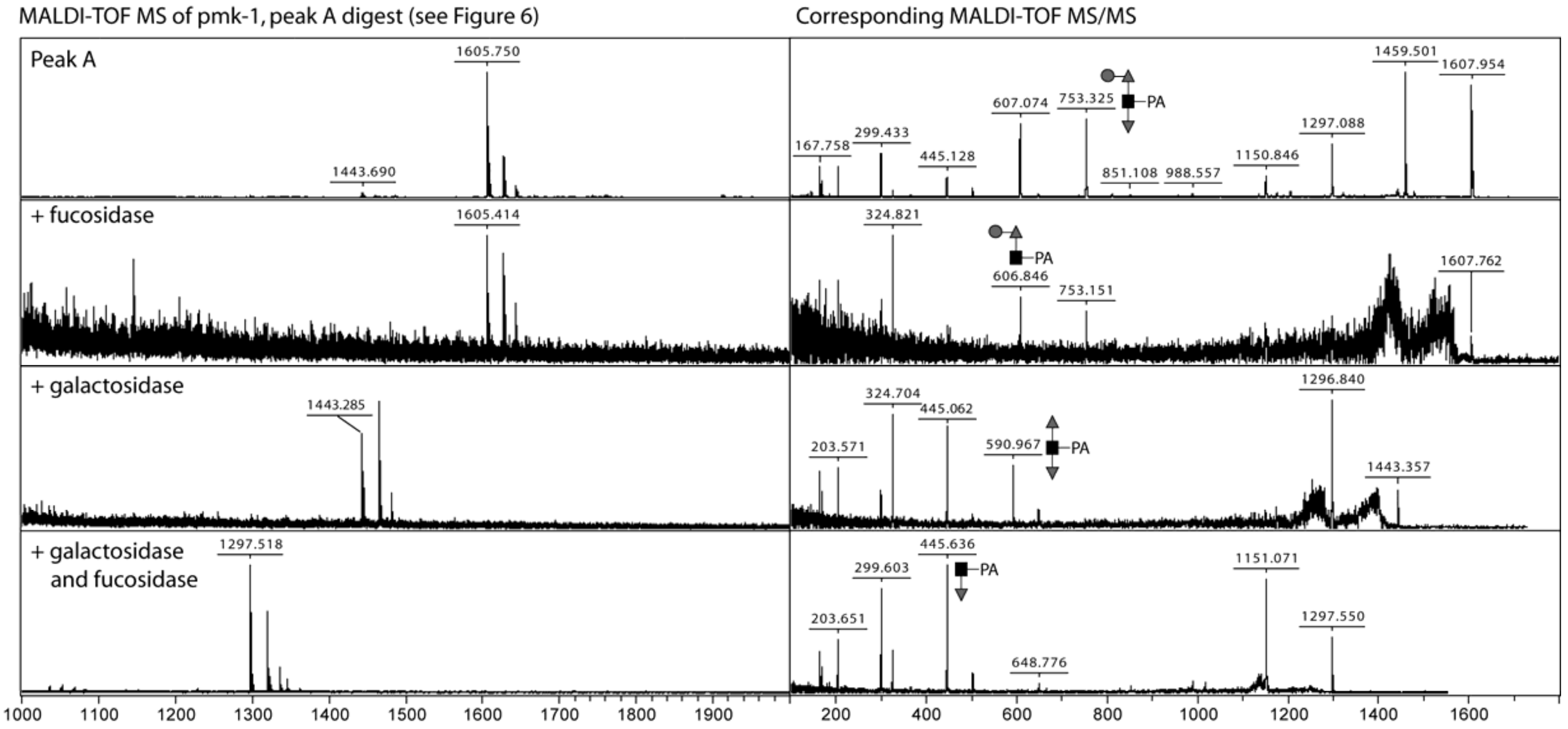

Figure S2. Mass spectrometric analysis of the $Hex_5dHex_2HexNAc_2$ glycan. The isolated fraction containing the $Hex_5dHex_2HexNAc_2$ N-glycan as well as aliquots of this glycan treated with either fucosidase alone, galactosidase alone or sequentially with galactosidase and fucosidase were analysed by MALDI-TOF MS; the spectra are annotated with the *m/z* values for the $[M+Na]^+$ species (left). The corresponding MALDI-TOF MS/MS fragmentation spectra are also shown and annotated with the putative structures of key diagnostic fragments (right).
