# Supplementary material for: Caenorhabditis elegans N-glycan Core β-galactoside Confers Sensitivity towards Nematotoxic Fungal Galectin CGL2
Source: PLoS Pathog. 2010 Jan 8;6(1):e1000717. doi: 10.1371/journal.ppat.1000717 (PMC2798750; doi:10.1371/journal.ppat.1000717)
Supplement: Figure S3 — The synthesis of Galβ1,4Fucα1,6GlcNAcβOC5H10NH2. Reaction conditions: a) AcOH, 50°C; b) Bu2SnO, toluene, reflux; c) BnBr, TBAI, 40°C; d) FmocCl, pyridine; e) K2CO3, Cl3CCN, DCM; f) N-(benzyl)-benzyloxycarbonyl-5-aminopentan-1-ol, TMSOTf, DCM, -15°C; g) HF-pyridine, THF; h) 4, DMTST, DTBMP, DCM, -10°C; i) Et3N; j) 2,3,4,6-tetra-O-benzoyl-β-D-galactopyranosyl trichloroacetimidate, TMSOTf, DCM, -10°C; k) ethylenediamine, nBuOH, reflux; l) Ac2O, pyridine; m) NaOMe, MeOH; n) Pd/C, H2, MeOH/H2O/AcOH. (0.12 MB PDF) [file ppat.1000717.s007.pdf]

Supporting Information: Supplementary Figure S3

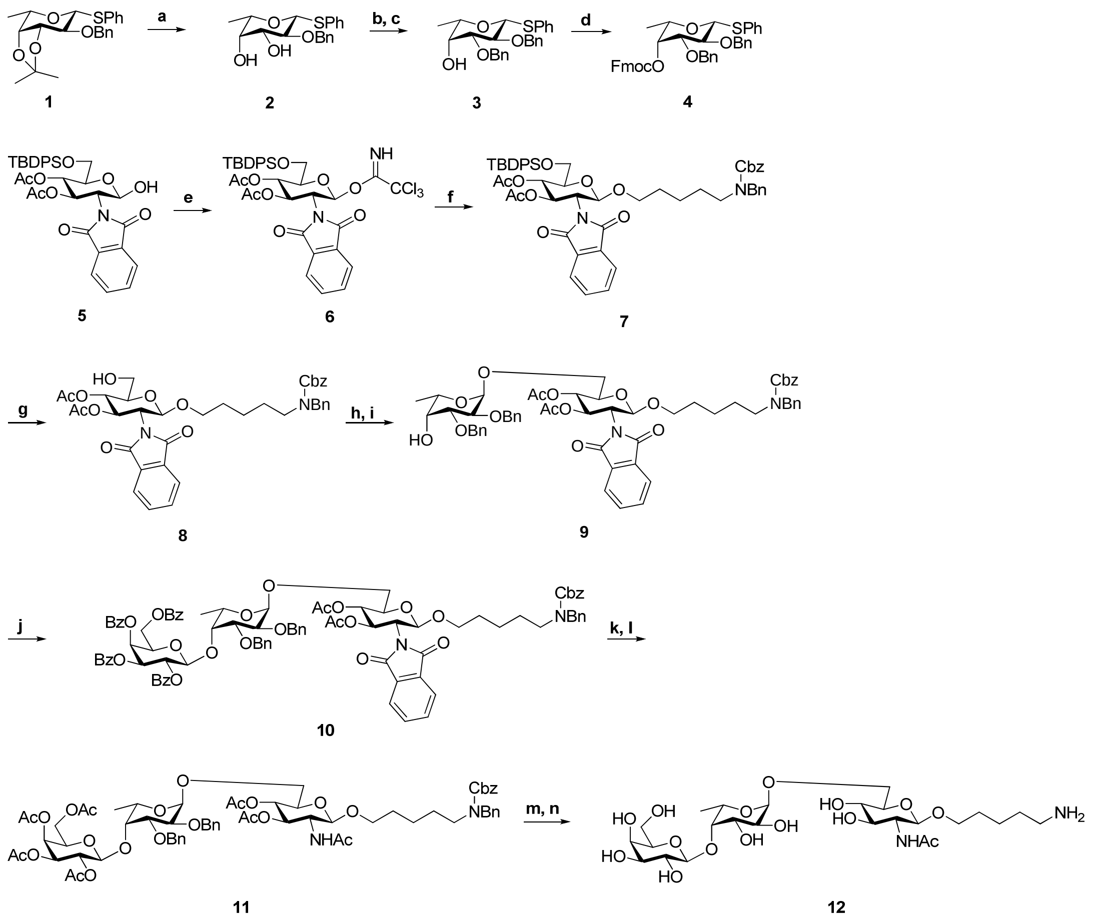

Figure S3. The synthesis of Galβ1,4Fucα1,6GlcNAcβOC$_5$H$_{10}$NH$_2$. Reaction conditions: a) AcOH, 50 °C; b) Bu_2SnO, toluene, reflux; c) BnBr, TBAI, 40 °C; d) FmocCl, pyridine; e) K_2CO_3, Cl_3CCN, DCM; f) N-(benzyl)-benzyloxycarbonyl-5-aminopentan-1-ol, TMSOTf, DCM, -15 °C; g) HF-pyridine, THF; h) **4**, DMTST, DTBMP, DCM, -10 °C; i) Et_3N; j) 2,3,4,6-tetra-*O*-benzoyl-*β*-D-galactopyranosyl trichloroacetimidate, TMSOTf, DCM, -10 °C; k) ethylenediamine, *n*BuOH, reflux; l) Ac_2O, pyridine; m) NaOMe, MeOH; n) Pd/C, H_2, MeOH/H_2O/AcOH.
